# Recent Advances in Nanoparticle-Based Strategies for Overcoming Hypoxia in Glioblastoma Photodynamic Therapy

**DOI:** 10.3390/ijms272110041

**Published:** 2026-11-09

**Authors:** Paweł Woźnicki, David Aebisher

**Affiliations:** 1 Doctoral School, Faculty of Medicine, Collegium Medicum, University of Rzeszów, 35-310 Rzeszów, Poland; pawelw@dokt.ur.edu.pl; 2 Department of Photomedicine and Physical Chemistry, Faculty of Medicine, Collegium Medicum, University of Rzeszow, 35-310 Rzeszów, Poland

**Keywords:** glioblastoma, photodynamic therapy, nanoparticles, hypoxia

## Abstract

Glioblastoma (GBM) remains one of the most aggressive primary brain tumours, and its hypoxic microenvironment represents a major barrier to effective photodynamic therapy (PDT). Reduced oxygen availability limits reactive oxygen species generation, while oxygen consumption during PDT may further aggravate tumour hypoxia and promote adaptive mechanisms associated with treatment resistance. A literature search covering January 2024 to August 2026 was conducted in PubMed, Scopus, and Web of Science using the Boolean query “PDT AND nanoparticles”. Original experimental studies investigating nanoparticle-based PDT in glioblastoma, with particular emphasis on strategies addressing hypoxia-related limitations, were considered eligible. Twelve studies were included in the qualitative synthesis and critically evaluated in terms of oxygen-modulating strategies, preservation of photodynamic activity under hypoxic conditions, therapeutic efficacy, and biosafety. The analysed nanoplatforms employ two principal strategies: direct alleviation of tumour hypoxia through local oxygen generation and partial circumvention of oxygen dependence through photochemical mechanisms that remain effective under reduced oxygen availability. Catalytic components including Pt, MnO_2_ and Fe_3_O_4_@BiFeO_3_ enable the conversion of endogenous H_2_O_2_ into oxygen, whereas type I photodynamic reactions provide an alternative means of maintaining ROS generation under hypoxic conditions. Beyond hypoxia modulation, multifunctional nanoplatforms integrate PDT with complementary therapeutic mechanisms, including photothermal therapy, redox disruption, ferroptosis, targeted protein degradation using PROTACs and immunomodulation. Several systems have demonstrated promising antitumour activity in experimental GBM models together with favourable preliminary biosafety profiles. However, substantial heterogeneity in experimental models, administration routes and irradiation protocols, as well as unresolved issues concerning long-term safety, blood–brain barrier penetration and clinical translation, remain important challenges. Overall, multifunctional nanoplatforms capable of modulating tumour hypoxia while simultaneously targeting additional mechanisms of GBM resistance represent a promising direction for the further development of PDT.

## 1. Introduction

Glioblastoma (GBM) is the most common primary malignant brain tumor in adults and one of the most aggressive tumors of the central nervous system. Despite multimodal treatment, the prognosis remains very poor, and the median survival is approximately 15–20 months [1,2,3]. The standard of care involves surgical resection with maximum safety, followed by radiation therapy and chemotherapy with temozolomide. Despite intensive treatment, GBM recurrences are common, and treatment options for recurrent disease are limited [3,4]. The effectiveness of treatment is primarily hampered by the infiltrative nature of the tumor and the significant heterogeneity of its cells [1,5]. Within a single tumor, populations with varying sensitivity to treatment may coexist, including cells with stem cell-like characteristics that are resistant to chemotherapy and radiation therapy [1,3]. Furthermore, the heterogeneity and plasticity of cells promote adaptation to therapeutic pressure and the development of resistance [3]. An additional barrier is the limited penetration of drugs across the blood–brain barrier, as well as the immunosuppressive microenvironment and metabolic plasticity of GBM cells [1,3,4]. As a result, current treatment strategies remain inadequate, highlighting the need to develop new treatment methods [3,6]. One promising approach is photodynamic therapy (PDT), which is based on the interaction of a photosensitizer, light of an appropriate wavelength, and molecular oxygen. Activation of the photosensitizer leads to the formation of reactive oxygen species (ROS) in Type I and Type II reactions, including free radicals and singlet oxygen. The resulting ROS can lead to the death of cancer cells, damage to tumor blood vessels, and the activation of an immune response [7,8]. A particularly promising direction for the development of PDT in GBM is its combination with nanotechnology. Nanoparticles can serve as carriers for photosensitizers, improving their solubility, stability, bioavailability, and selectivity of delivery to tumor tissue, as well as facilitating the crossing of the blood–brain barrier. Multifunctional nanoplatforms can, moreover, not only transport the photosensitizer but also modify the tumor microenvironment [6]. This is particularly important in GBM, as hypoxia is one of the characteristic features of its microenvironment and a significant factor in the tumor’s aggressiveness. It results, among other things, from rapid tumor growth, which outpaces the development of the vascular system [9]. Despite its high degree of vascularization, microcirculation in GBM remains inefficient, which contributes to the development of areas of severe hypoxia and necrosis [10]. Oxygen deprivation leads to the stabilization of HIF-1 and the activation of hypoxia-responsive genes, including VEGF [9]. HIF-1 also regulates processes related to the metabolism and survival of cancer cells [6,11]. However, the new blood vessels that form are often abnormal and dysfunctional, which further exacerbates hypoxia [3]. Hypoxia also promotes anaerobic glycolysis, the maintenance of stem cell characteristics, and resistance to treatment [6,12], and vascular disorders can lead to cyclic hypoxia [13]. It also affects the immune microenvironment of GBM and may further limit drug delivery to the tumor [14]. Because PDT depends on oxygen, the hypoxic microenvironment of GBM is one of the main limitations of this therapy. Oxygen deprivation reduces ROS production, while PDT itself consumes oxygen, which may exacerbate tumor hypoxia [6]. Vascular damage and impaired perfusion caused by PDT may also contribute to a further worsening of hypoxia [11]. A decrease in oxygen availability can activate HIF-1, and oxidative stress can trigger antioxidant defense mechanisms, promoting the survival of some cells after treatment [6,11]. For this reason, nanoparticle systems capable of locally delivering or generating oxygen and modifying the hypoxic tumor microenvironment are of particular interest [6,15]. Combining such nanoplatforms with PDT can simultaneously improve photosensitizer delivery and counteract hypoxia, representing a particularly promising direction for the development of GBM therapies.

This review critically evaluates nanoparticle-based strategies developed to overcome hypoxia-associated limitations of photodynamic therapy in glioblastoma. A literature search was conducted in PubMed, Scopus, and Web of Science, covering publications from January 2024 to August 2026, using the Boolean query “PDT AND nanoparticles”. Original experimental studies investigating glioblastoma and employing nanostructured PDT-based therapeutic platforms were considered eligible. Review articles, studies unrelated to glioblastoma, and studies that did not investigate PDT or did not incorporate a nanostructured therapeutic system were excluded. Following application of these eligibility criteria, 12 original studies were included in the qualitative synthesis. The selected studies were critically assessed with particular emphasis on oxygen-generating mechanisms, changes in oxygen availability within biological systems, preservation of photodynamic activity under hypoxic conditions, therapeutic efficacy, and biosafety. Particular attention was paid to distinguishing experimentally demonstrated effects from proposed mechanisms and identifying methodological limitations and research gaps relevant to further preclinical development. The specified publication period applies to the original experimental studies included in the qualitative synthesis, whereas earlier publications were cited where necessary to provide scientific background and mechanistic context.

## 2. Architecture and Functional Components of Nanoplatforms

### 2.1. Organisation of the Substrate and the Photoactive Module

The architecture of the nanoplatform determines how the photosensitiser is positioned in relation to the carrier and the other functional components, thereby influencing its state of aggregation, the availability of reaction substrates and the course of photochemical processes [16,17]. Lu et al. [18] They developed porphyrin metal-organics frameworks (MOFs) in which the chromophores were incorporated into an ordered, porous scaffold, ensuring their spatial separation and limiting aggregation and self-quenching. A similar concept was applied by Hu et al. [19] in Pt@PCN-222(Mn)-PEG, where the zirconium nodes and Mn(III)-containing porphyrin ligands simultaneously form both the carrier scaffold and the photoactive module. The structure is complemented by platinum nanoparticles and a surface layer of poly(ethylene glycol) (PEG). A different arrangement was presented by Chen et al. [15], by developing a PLCNP with a CaO_2_ core surrounded by a lipoprotein layer containing DOPA and DMPC lipids, a porphyrin–lipid conjugate and apolipoprotein E3. The positioning of the chromophores within the shell was linked to the breakdown of the core and the reorganisation of the nanostructure, thereby limiting their mutual quenching. Spatial separation of the components was also employed by Jiang et al. [20] in the SOP MN system, in which pH-sensitive PPT7 nanoparticles, containing the PEG-b -PDPA-PPa conjugate and the prodrug OT7, were embedded in a rapidly dissolving layer of hyaluronic acid, whilst BSA–MnO_2_ was embedded in a more slowly degrading layer of photocross-linked methacrylated hyaluronic acid. The bilayer structure of the microneedles allows for the differentiation of the release kinetics of both components.

In hybrid platforms, the photoactive module is integrated with inorganic or carbon-based materials. Dash et al. [21] They developed rGOQD/IR820/MnO_2_/Q/CPP, in which the IR820 dye was covalently bound to reduced graphene quantum dots, and further functionalisation included PEGylated lipids, albumin-stabilised MnO_2_, quercetin and a cell-penetrating peptide. In H-CeO_2_@Ce6@PDA@GOx, described by Süngü Akdogan et al. [22], Chlorine E6 was adsorbed onto hollow mesoporous CeO_2_ nanoparticles, which were then coated with polydopamine and functionalised with glucose oxidase. Li et al., on the other hand, ref. [23] They synthesised Fe_3_O_4_@PDA@Ru using a magnetite core, a poly-dopamine and PEG shell, and an immobilised ruthenium(II) complex, [Ru(bpy)_2_(PYIP)]Cl_2_, acting as a photosensitiser.

A separate group consists of nanoplatforms in which the photoactivity stems directly from the properties of the inorganic material, without the involvement of a conventional organic photosensitiser. In Bi_2_S_3_/Ti_3_C_2_-TPP, Jiang et al. [24] They combined bismuth sulphide with the MXene Ti_3_C_2_, producing a heterostructure whose phase boundary promotes the separation of photo-excited charge carriers. The surface was modified with PEG and triphenylphosphonium groups. Zhao et al. employed a more complex biomimetic architecture [25] in FBFO@HM@aOPN, where the Fe_3_O_4_@BiFeO_3_ core is surrounded by a hybrid membrane composed of M1 macrophage vesicles and bacterial outer membrane vesicles, further functionalised with an anti-osteopontin antibody.

Among organic platforms, control of the molecular structure and self-organisation of photosensitisers is also of significant importance [26]. Zhang et al. [27] They utilised the TTTMN luminogen, which exhibits aggregation-induced emission (AIE), encapsulated within nanoparticles formed from PEGylated phospholipids and functionalised with angiopoietin-2, to create TTTMN NPs-Ang2. The donor–acceptor structure and the twisted geometry of the chromophore help to preserve its photochemical activity in the aggregated state. In the LTPA, Zhang et al. [28] Instead, they used a self-assembling conjugate of the photosensitiser LT linked to angiopep-2 via a PEG linker. Another approach was presented by Zou et al. [29], by developing JSK@PEG-IR820, in which a nitric oxide donor is encapsulated in a pH-sensitive carrier containing a covalently bound IR820 dye and diisopropylamine segments. A simpler structure was described by Caldas et al. [30], using natural melanin nanoparticles derived from Sepia officinalis ink for the non-covalent immobilisation of rhodamine B isothiocyanate.

The solutions presented include the incorporation of chromophores into ordered frameworks, their immobilisation on the surface or within a carrier, the self-organisation of photosensitisers, and the direct utilisation of the photoactivity of inorganic materials. These differences determine the organisation of the photoactive module and form the basis for the further functionalisation of nanoplatforms intended for GBM therapy.

### 2.2. Targeting and Delivery

**Figure 1:**
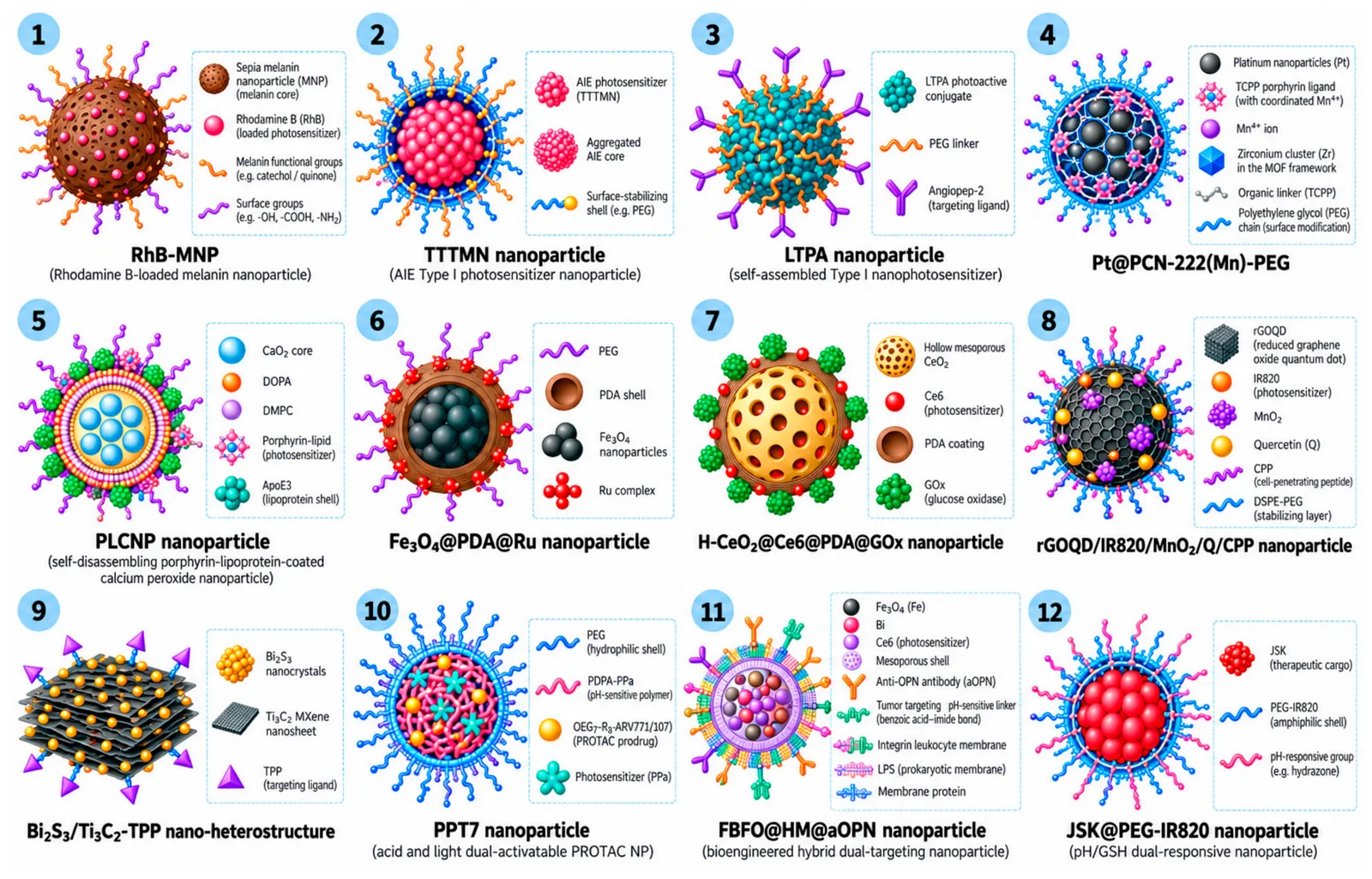
Schematic representation of nanoparticle-based photodynamic therapeutic platforms investigated for glioblastoma.

The efficacy of nanoplatforms in photodynamic therapy (PDT) for glioblastoma multiforme (GBM) depends not only on their photochemical properties, but also on the ability to deliver the photosensitiser to the tumour tissue and ensure its appropriate cellular localisation. Significant limitations remain in the form of the blood–brain barrier (BBB) and the heterogeneous permeability of the blood–tumour barrier (BBTB), which hinder the transport and uniform distribution of nanocarriers [31,32]. The nanoplatforms analysed utilised various strategies to enhance transport across the blood–brain barrier (BBB), accumulation in the GBM and cellular uptake. In *TTTMN NPs-Ang2* and *LTPA*, angiopep-2 was used, which interacts with the *LRP1* receptor, the increased expression of which has been confirmed in GBM tissues derived from patients [33,34]. Functionalisation with this peptide was intended to facilitate both the crossing of the blood–brain barrier and the uptake of the nanoplatforms by tumour cells [27]. A different strategy was employed in *PLCNP*, utilising a lipoprotein coat containing apolipoprotein E3 (ApoE3), which interacts with lipoprotein receptors, including *LDLR* and *LRP1* (Figure 1). Increased expression of *LDLR* has been reported in human GBM tissues, whilst the uptake of *PLCNP* by glioma-initiating cells has been linked primarily to macropinocytosis [15,35].

In rGOQD/IR820/MnO_2_/Q/CPP, a cell-penetrating peptide (CPP) was used to enhance the internalisation of the nanoparticles; the authors also suggested the involvement of the transferrin receptor (TfR1), the overexpression of which was confirmed in GBM samples [21,36]. A biomimetic strategy was, however, employed in FBFO@HM@aOPN, whose hybrid macrophage-bacterial coating contains the α4β1 integrin, which interacts with VCAM-1, which is overexpressed in GBM vessels [25,37]. An additional functional component was an antibody directed against osteopontin (OPN), which was found to be overexpressed in GBM, particularly in the mesenchymal subtype. Neutralisation of OPN was thought to promote tumour penetration by modulating its microenvironment [25,38].

A distinct approach is subcellular targeting, as employed in Bi_2_S_3_/Ti_3_C_2_-TPP. The triphenylphosphonium (TPP) groups are not responsible for the specific recognition of GBM cells, but rather promote the accumulation of the nanoplatform in the mitochondria following its internalisation, by utilising the negative potential of the mitochondrial membrane [24]. Other nanoplatforms, which are not equipped with specific targeting ligands, utilise the physicochemical properties of the carriers and passive distribution mechanisms. An alternative to systemic delivery is the local administration of therapeutic agents using SOP MN microneedles, which avoids the need for transport across the blood–brain barrier [20].

### 2.3. Additional Active Ingredients

In order to enhance the efficacy of PDT, some of the nanoplatforms under investigation were equipped with additional therapeutic components, enabling simultaneous modulation of cancer cell survival mechanisms, metabolism and the GBM microenvironment. One such strategy was employed by Jiang et al. [20] in the MN SOP, by introducing the OT7 prodrug derived from ARV-771, a protein degrader from the BET (bromodomain and extraterminal domain) family. These proteins are involved in the regulation of transcription and the proliferation of cancer cells, and increased expression of BRD2 and BRD4, as well as the role of BRD4 in cell cycle progression, has been confirmed in GBM studies [39]. ARV-771 is a PROTAC molecule which, by recruiting the ubiquitin ligase VHL, induces proteasomal degradation of BET proteins, including BRD2, BRD3 and BRD4 [40]. The SOP MN utilises its OT7 derivative, which contains a linker susceptible to cleavage by singlet oxygen, enabling the release of the active degradator following photoactivation of pyropheophorbide a [20].

Dash et al. utilised a different mechanism to enhance the therapy [21], by incorporating quercetin into rGOQD/IR820/MnO_2_/Q/CPP. This natural flavonoid may inhibit the expression of heat shock proteins, in particular HSP70, which are involved in protecting cancer cells from thermal damage. Previous studies have demonstrated quercetin’s ability to modulate the HSF1/HSP70 pathway and enhance the efficacy of thermal ablation of solid tumours [41]. In the nanoplatform under investigation, it was therefore used to limit thermotolerance and enhance the efficacy of photothermal therapy (PTT). JSK in JSK@PEG-IR820 serves a different function. It is a prodrug that releases nitric oxide (NO) upon activation involving glutathione (GSH). The resulting NO can react with the superoxide anion radical (O_2_^•−^), leading to the formation of the highly cytotoxic nitrosyl radical (ONOO^−^) [29]. In the case of H-CeO_2_@Ce6@PDA@GOx, glucose oxidase (GOx) was used—an enzyme that catalyses the oxidation of glucose, whose therapeutic function has been linked to limiting the availability of energy substrates to GBM cells [22].

An additional therapeutic effect in some of the nanoplatforms is provided by the conversion of radiation energy into heat. The photothermal function is performed, amongst others, by carbon-based materials in rGOQD/IR820/MnO_2_/Q/CPP, polydopamine in Fe_3_O_4_@PDA@Ru and H-CeO_2_@Ce6@PDA@GOx, the Bi_2_S_3_/Ti_3_C_2_ heterostructure, and the LT chromophore in LTPA, enabling the combination of PDT with PTT [21,22,23,24,28].

Zhao et al. adopted a different strategy [25] in FBFO@HM@aOPN, combining PDT with immunomodulation. The anti-OPN antibody neutralises osteopontin, a protein involved in macrophage recruitment and the formation of the immunosuppressive microenvironment of GBM [38]. In turn, bacterial outer membrane vesicles (OMVs), which form part of the nanoplatform’s coating, deliver immunostimulatory signals that promote the reprogramming of macrophages towards an anti-tumour phenotype. In post-operative studies, an anti-PD-1 antibody was also used as a separate combination immunotherapy, rather than as a component of the nanoplatform itself [25].

## 3. Strategies for Mitigating the Effects of Hypoxia

### 3.1. Increasing the Local Availability of Oxygen

Increasing local oxygen availability is one of the strategies for counteracting the limitations of PDT resulting both from hypoxia in the tumour microenvironment and from the additional consumption of O_2_ during the photoactivation of the photosensitiser. This phenomenon was documented by Sitnik et al. [42], demonstrating, in a mouse RIF tumour model, a sharp drop in oxygen partial pressure (pO_2_) within the first min of irradiation, as well as the dependence of changes in oxygenation on the power density of the radiation and interruptions in exposure. These observations justify the design of nanoplatforms capable of increasing the availability of O_2_ directly in the vicinity of the photosensitiser whilst photodynamic reactions are taking place.

One of the most commonly used methods for local oxygen generation is the decomposition of hydrogen peroxide (H_2_O_2_), a natural product of cellular oxidative processes. The significance of this compound in GBM biology was demonstrated by Lei et al. [43], noting that hypoxia promotes the accumulation of H_2_O_2_, whilst activation of HIF-1α increases the expression of glutathione peroxidase 1 (GPx1), which is involved in its removal and in maintaining redox balance. The presence of H_2_O_2_ in glioma tissue is also confirmed by quantitative measurements. Yin et al. [44], Using surface-enhanced Raman spectroscopy (SERS), they determined mean H_2_O_2_ concentrations of 41.88 ± 10.92 µM in orthotopic U251 tumours and 68.54 ± 4.51 µM in IDH1-WT GL261 tumours. The authors also carried out measurements in freshly resected glioma tissues from patients, confirming that H_2_O_2_ levels varied depending on the molecular characteristics of the tumour. By way of comparison, in studies of normal rat brain tissue using the microdialysis method, extracellular H_2_O_2_ concentrations of approximately 3 µM were measured in the ventrolateral medulla and 5 µM in the hippocampus [45].

The presence of H_2_O_2_ in the GBM microenvironment forms the basis for the design of nanoplatforms that utilise its catalytic or redox decomposition for the local production of O_2_. This strategy has been applied, amongst others, in Pt@PCN-222(Mn)-PEG, SOP MN, rGOQD/IR820/MnO_2_/Q/CPP and FBFO@HM@aOPN, utilising various inorganic components capable of initiating H_2_O_2_ transformations [19,20,21,25].

In the analysed nanoplatforms utilising H_2_O_2_ as a substrate for oxygen-generating reactions, inorganic components exhibiting catalase-like or photocatalytic activity were employed. Depending on the material’s properties, O_2_ release may occur via catalase-like disproportionation of H_2_O_2_ (2H_2_O_2_ → 2H_2_O + O_2_), its redox reactions with metal oxides, or photo-induced oxidation processes. The underlying principles of these strategies were previously outlined by Zhang et al. [46], using platinum nanoparticles immobilised on porphyrin metal-organics frameworks (MOFs), and Prasad et al. [47], who used albumin-stabilised MnO_2_ nanoparticles to regulate oxygenation of the tumour microenvironment.

Hu et al. employed a strategy based on the catalase-like activity of platinum [19] in Pt@PCN-222(Mn)-PEG. Pt nanoparticles embedded in a porphyrin MOF catalyse the decomposition of H_2_O_2_ into H_2_O and O_2_, increasing the local availability of oxygen, which is then utilised by the photoactive Mn-TCPP ligands to generate singlet oxygen (^1^O_2_). Concurrently, Mn(III) centres participate in reactions leading to the consumption of glutathione (GSH) and the reduction of Mn(III) to Mn(II), thereby limiting the cells’ ability to neutralise ROS. In this structure, the functions of oxygen generation and redox balance regulation are thus assigned to distinct metal centres.

MnO_2_ employs a different mechanism; in an acidic environment, it can react with H_2_O_2_ whilst simultaneously releasing O_2_ and reducing Mn(IV) to Mn(II) (MnO_2_ + H_2_O_2_ + 2H^+^ → Mn^2+^ + O_2_ + 2H_2_O). These properties have been utilised in SOP MN and rGOQD/IR820/MnO_2_/Q/CPP, using BSA–MnO_2_ nanoparticles stabilised with albumin [20,21]. In SOP MN, the oxygen-generating module is embedded within an inner, more slowly degrading layer of microneedles, ensuring the gradual release of BSA–MnO_2_ following the prior delivery of PPT7 nanoparticles. The resulting O_2_ is utilised during the photoactivation of pyropheophorbid a, leading to the generation of ^1O_2_, which also participates in the cleavage of the OT7 prodrug linker [20]. In contrast, in gGOQD/IR820/MnO_2_/Q/CPP, the MnO_2_ component has been integrated into a support containing IR820, enabling localised oxygen generation in the immediate vicinity of the photosensitiser [21].

Süngü Akdogan et al. also utilised the catalase-like activity of cerium oxide [22] in H-CeO_2_@Ce_6_@PDA@GOx. The redox properties of CeO_2_, associated with the transitions between Ce(III) and Ce(IV), enable the decomposition of H_2_O_2_ with the release of O_2_. Unlike systems utilising only the H_2_O_2_ present in the tumour microenvironment, the nanoplatform has been additionally equipped with glucose oxidase (GOx), which catalyses the oxidation of glucose whilst simultaneously consuming O_2_ and producing H_2_O_2_. The resulting hydrogen peroxide can then undergo catalase-like decomposition on the CeO_2_ surface, forming a cascade enzymatic-catalytic system in which part of the oxygen consumed by GOx is re-released and can participate in the photoactivation of chlorin e6.

Another pathway for the conversion of H_2_O_2_ was presented by Zhao et al. [25], by developing FBFO@HM@aOPN with a Fe_3_O_4_@BiFeO_3_ photocatalytic core. Under the influence of radiation, electron–hole (e^−^/h^+^) pairs are generated in the heterostructure; their separation enables the charge carriers to participate in redox reactions. Photogenerated holes (h^+^) can oxidise H_2_O_2_ to release O_2_, whilst electrons participate in the reduction of oxygen to the superoxide anion radical (O_2_^•−^). Concurrently, iron centres present in the structure can catalyse Fenton-type reactions involving H_2_O_2_, leading to the production of hydroxyl radicals (^•^OH). In this way, the photoactivity of the heterostructure has been linked to H_2_O_2_ transformations, encompassing both the release of oxygen and the generation of radical ROS.

An alternative to utilising H_2_O_2_ present in the tumour microenvironment is to incorporate a chemical oxygen source into the nanoplatform. One such material is calcium peroxide (CaO_2_), which releases H_2_O_2_ in an aqueous environment, subsequently converting to H_2_O and O_2_. Sheng et al. demonstrated the potential for utilising this mechanism in PDT [48], by developing CaO_2_ nanoparticles with a polymer coating that allows the release of oxygen to be regulated depending on the pH.

This strategy was employed by Chen et al. [15] in PLCNP, by combining a CaO_2_ core with a lipoprotein shell containing a porphyrin photosensitiser. Following the internalisation of the nanoparticles, the acidic environment of the lysosomes promotes the decomposition of CaO_2_, leading to the release of H_2_O_2_ and the subsequent formation of O_2_. The disintegration of the core and the increase in osmotic pressure accompanying these changes facilitate the destabilisation of the lysosomes and the release of porphyrin components into the cytoplasm. At the same time, the presence of the CaO_2_ core and the subsequent reorganisation of the nanostructure limit the mutual quenching of chromophores, combining local oxygen delivery with the activation of the photosensitiser’s photo-optical properties.

A separate strategy for increasing local oxygen availability is the photocatalytic oxidation of water, which utilises photogenerated charge carriers to initiate reactions leading to the release of O_2_. This approach was employed by Jiang et al. [24] in the Bi_2_S_3_/Ti_3_C_2_-TPP nanoplatform, utilising a semiconductor heterostructure with a Schottky junction at the Bi_2_S_3_–Ti_3_C_2_ phase boundary. Under irradiation at a wavelength of 808 nm, photogenerated electrons and holes are separated, thereby limiting their recombination. According to the mechanism proposed by the authors, the holes participate in the oxidation of H_2_O with the evolution of O_2_ (2H_2_O + 4h^+^ → O_2_ + 4H^+^), whilst the electrons enable the reduction of oxygen to the superoxide anion radical (O_2_^•−^). Its subsequent transformations may lead to the formation of H_2_O_2_ and hydroxyl radicals (^•^OH), linking local oxygen production with type I photodynamic reactions.

An emerging direction in the development of hypoxia-oriented nanotherapeutic platforms involves single-atom nanozymes, in which isolated metal atoms are stabilised within defined coordination environments and act as catalytic centres. Compared with conventional metal nanoparticles, this architecture offers opportunities to increase metal-atom utilisation and modulate catalytic activity through control of the local coordination structure. Wang et al. [49] developed a Ru single-atom nanozyme integrated with chlorin e6, in which catalase-like decomposition of H_2_O_2_ provided O_2_ for photosensitiser-mediated singlet oxygen generation. Subsequently, Wang et al. [50] demonstrated that atomically dispersed Pt sites incorporated into a metal–organic framework exhibited greater catalase-like activity than a corresponding Pt nanoparticle-based system, enhancing oxygen generation and PDT-associated responses. More recently, Li et al. [51] combined a Pt single-atom nanozyme with a photosensitiser-containing platform and PD-L1-directed immunomodulation, illustrating the potential integration of oxygen-generating catalysis with complementary antitumour mechanisms.

Single-atom engineering may also enable alternative oxidative pathways. For example, Liu et al. [52] developed an O-coordinated Fe–N_4_ catalyst capable of generating singlet oxygen through H_2_O_2_-dependent catalytic reactions under hypoxic conditions without external irradiation. Although this represents catalytic therapy rather than conventional PDT, it illustrates how the coordination environment of isolated metal centres can influence ROS-generation pathways. These findings suggest opportunities for designing future hypoxia-adapted photodynamic systems; however, they should not be interpreted as evidence of therapeutic efficacy in GBM. The practical advantages of single-atom nanozymes will require validation under biologically relevant H_2_O_2_ concentrations, direct assessment of oxygen availability and photochemical activity during irradiation, and evaluation of catalyst stability, intracranial delivery and long-term safety.

### 3.2. Reducing the Dependence of Performance on Oxygen Availability

An alternative to increasing the local availability of oxygen is the design of nanoplatforms that utilise photochemical pathways less dependent on its concentration. Unlike type II PDT, which is based on the transfer of excitation energy directly to O_2_ and the generation of singlet oxygen (^1^O_2_), the type I mechanism involves the transfer of electrons or hydrogen atoms between the excited photosensitiser and the surrounding substrates, leading to the formation of reactive radical species [53,54]. These processes may reduce the dependence of PDT on the availability of molecular oxygen, although they do not always eliminate it entirely, as the formation of certain ROS, including the superoxide anion radical (O_2_^•−^), still requires the involvement of O_2_.

One strategy for promoting Type I reactions is the appropriate design of the electronic structure of photosensitisers. Zhang et al. [28] They applied this approach in the LTPA nanoplatform, utilising an LT chromophore with a donor–acceptor (D–A) structure, containing a triphenylamine group acting as an electron donor and a thiopyril heterocyclic acceptor. The introduction of the triphenylamine group was intended to promote an intersystem crossing (ISC), lower the reduction potential and facilitate electron transfer leading to the formation of O_2_^•−^. A different D–A system was used by Zhang et al. [27] in TTTMN NPs-Ang2. The TTTMN photosensitiser contains a triphenylamine moiety acting as an electron donor, a thiophene moiety involved in electron donation and acting as a π-bridge, and two cyano groups acting as acceptors. This organisation of the electronic structure facilitates charge transfer processes and electron transfer reactions leading to the formation of radical ROS, including O_2_^•−^ and ^•^OH. Furthermore, the aggregation-induced emission (AIE) properties enable the use of the photosensitiser in the form of nanoparticles, limiting the aggregation-induced quenching characteristic of many conventional chromophores and promoting the preservation of photochemical activity in the aggregated state [55,56].

A different strategy was presented by Zou et al. [29], by developing JSK@PEG-IR820, in which the promotion of Type I photochemistry is combined with the regulation of intracellular redox equilibrium. The diisopropylamine groups present in the polymeric carrier enable a response to an acidic environment by destabilising the nanostructure and, at the same time, in accordance with the proposed mechanism, facilitate electron transfer to the IR820 dye and the generation of O_2_^•−^ following its photoactivation. The JSK prodrug released from the nanoplatform is activated in the presence of glutathione (GSH), leading to its consumption and the release of nitric oxide (NO). Reduced GSH availability may impair the cellular mechanisms responsible for neutralising the oxidative products formed during PDT [57], In contrast, the reaction of NO with O_2_^•−^ leads to the formation of the highly reactive peroxynitrite (ONOO^−^) [58]. This strategy therefore combines the promotion of the PDT radical pathway with the suppression of cellular antioxidant defences and the sary production of reactive nitrogen species.

Electron transfer reactions have also been utilised in inorganic semiconductor nanoplatforms. Jiang et al. [24] They utilised a Bi_2_S_3_/Ti_3_C_2_ heterostructure, in which the interface between bismuth sulphide and the MXene Ti_3_C_2_ promotes the separation of photogenerated electrons and holes, thereby limiting their recombination. Electrons can participate in the reduction of O_2_ to O_2_^•−^, whilst oxidation reactions involving photogenerated holes can lead to the formation of ^•^OH. The nanoplatform thus combines Type I radical photochemistry with Type II reactions and the photocatalytic oxygen production described earlier. A related concept was employed by Zhao et al. [25] in FBFO@HM@aOPN, utilising a photoactive Fe_3_O_4_@BiFeO_3_ core. The separation of photogenerated electron–hole pairs enables electrons to participate in the reduction of O_2_ to O_2_^•−^ and holes to participate in oxidation reactions leading to the formation of ^•^OH. In addition, the iron centres can catalyse the conversion of H_2_O_2_ in Fenton-like and peroxidase-like reactions, providing an additional source of reactive oxidation products, independent of the direct excitation of the photosensitiser [25,59,60]. In both structures, therefore, the reduction in oxygen dependence is based on the co-occurrence of photochemical and redox pathways with differing O_2_ requirements.

Caldas et al. adopted a different approach [30] in RhB-MNPs, using rhodamine B immobilised on natural melanin nanoparticles. Rhodamine B may participate in electron transfer reactions with appropriate substrates, characteristic of the type I mechanism [61]. At the same time, one possible mechanism for its photoactivity is the transfer of excitation energy to molecular oxygen, leading to the generation of ^1^O_2_ via a type II mechanism. The co-occurrence of both pathways has also been proposed for RhB-MNPs [30]. Unlike systems containing specially designed Type I photosensitisers, this strategy is not based on modifying the electronic structure of the chromophore to promote electron transfer, but on utilising the existing photochemical properties of rhodamine B in combination with a nanocarrier that enhances its delivery to cells.

The solutions presented therefore include modifying the electronic structure of photosensitisers to promote Type I reactions, limiting the cellular neutralisation of ROS, utilising semiconductor heterostructures, and combining photodynamic processes with additional redox reactions. These strategies do not lead to the complete elimination of the dependence of the reaction on O_2_, but are intended to enable the use of alternative reaction pathways when O_2_ is in limited supply.

## 4. Assessment of the Reduction in Hypoxia and Oxygen Dependence

An assessment of strategies to mitigate the effects of hypoxia on PDT requires a distinction to be made between the oxygen-generating capacity of nanoplatforms, their impact on the oxygenation of the biological environment, and their ability to maintain photochemical activity under hypoxic conditions [62,63]. These are related but distinct aspects of the process, the significance of which depends on the mechanism employed in a given nanoplatform. In oxygen-generating systems, it is crucial to determine whether the chemical transformations lead to an increase in the availability of O_2_ for photodynamic reactions [42], whereas in strategies based on Type I photochemistry and alternative radical pathways—to what extent does the generation of reactive oxygen species persist under conditions of limited oxygenation [54]. This classification allows for an analysis of both approaches in terms of their respective mechanisms and experimental results, without imposing the same evaluation criteria on all nanoplatforms.

### 4.1. Oxygen-Producing Capacity and the Balance of Oxygen-Producing Processes

Any assessment of the oxygen-producing capacity of nanoplatforms must take into account not only their demonstrated catalytic or photocatalytic activity, but also the concentration of available substrates, the reaction conditions and concurrent processes that consume O_2_ [64,65]. A key factor determining the course of oxidising reactions is the local availability of H_2_O_2_, the concentration of which may limit the rate of reactions catalysed by nanomaterials [64]. The study by Yin et al. provides an important point of reference [44], in which H_2_O_2_ concentrations of 41.88 ± 10.92 µM were measured in orthotopic U251 tumours and 68.54 ± 4.51 µM in IDH1-WT GL261 tumours. Although these values allow an assessment of the biological rationale for the substrate concentrations used in the experiments, they do not directly reflect the amount of H_2_O_2_ available to the nanocatalysts in the extracellular space. This is because its local availability is limited by competitive consumption by endogenous antioxidant systems, including glutathione peroxidase GPx1, the expression of which in GBM increases in response to hypoxia [43]. An additional factor influencing the reaction balance is the co-occurrence of catalase-like and peroxidase-like activity in certain nanozymes, leading to the competitive utilisation of H_2_O_2_ in processes yielding different reaction products [64]. In Pt@PCN-222(Mn)-PEG, the catalase-like activity of the platinum nanoparticles was assessed by measuring the concentration of dissolved O_2_ during the reaction with H_2_O_2_. Hu et al. [19] They used substrate concentrations of 1.25, 2.5, 5 and 10 µM, recording a concentration-dependent increase in oxygen content over a 10-min period. In addition, at 100 µM H_2_O_2_, the formation of gas bubbles was observed. These studies confirm the oxygen-generating activity of the material even at H_2_O_2_ concentrations lower than those specified in GBM models. However, the rate of O_2_ production under conditions of continuous biological substrate renewal, or its dependence on competing redox reactions occurring in the tumour microenvironment, has not been determined. According to the stoichiometry of catalase-like H_2_O_2_ disproportionation (2H_2_O_2_ → 2H_2_O + O_2_), the complete conversion of 10 µM of substrate could theoretically yield a maximum of 5 µM of O_2_ [66]. However, this value does not reflect the actual availability of oxygen during PDT, which also depends on the continuous supply of H_2_O_2_, the transport of the O_2_ produced, and its simultaneous consumption in photochemical reactions [65].

MnO_2_ exhibits different properties; in an acidic environment, it reacts with H_2_O_2_, leading to the release of O_2_ and the simultaneous reduction of Mn(IV) to Mn(II) [47,67]. In contrast to the catalytic decomposition of H_2_O_2_ into H_2_O and O_2_ involving Pt nanoparticles, the reaction of MnO_2_ in an acidic environment leads to the reduction of Mn(IV) to Mn(II) and the gradual consumption of the oxide material. The amount of oxygen released therefore depends both on the availability of H_2_O_2_ and on the amount of reactive MnO_2_ [47,67].

In SOP MN Jiang et al. [20] they assessed the ability of BSA–MnO_2_ nanoparticles to produce O_2_ in the presence of 100 µM H_2_O_2_, comparing the course of the reaction at pH 7.4 and 6.5. Direct measurements of dissolved oxygen revealed a clear dependence of the process on the acidity of the environment. At pH 6.5, the O_2_ concentration rose sharply in the first few min of the reaction, after which it gradually decreased, whilst in a neutral environment it remained at a significantly lower level. The area under the curve of O_2_ concentration changes over 15 min was approximately 54.6 at pH 6.5, compared with approximately 18.1 at pH 7.4, which corresponds to an approximately threefold greater cumulative measured response in the acidic environment. These results confirm the significant effect of acidification on the oxygen-generating activity of BSA–MnO_2_ at an H_2_O_2_ concentration close to the values determined in GBM models [44]. However, the authors did not specify the molar conversion efficiency of H_2_O_2_ to O_2_ or the degree of MnO_2_ consumption, which makes it impossible to draw up a complete reaction balance. Furthermore, the measurements were carried out on a BSA–MnO_2_ suspension rather than on the complete SOP MN microneedle system. The prolonged release of nanoparticles from the inner layer of the microneedles, demonstrated in separate experiments, therefore does not directly confirm that O_2_ production continues for the same period.

Dash et al. employed significantly less representative conditions [21] in the rGOQD/IR820/MnO_2_/Q/CPP studies. In the experiments documenting O_2_ release, 40 mM H_2_O_2_ was used, a concentration approximately 580–950 times higher than the values determined by Yin et al. in orthotopic glioma models [44]. The authors observed the formation of gas bubbles following the addition of H_2_O_2_ to a nanocomposite containing MnO_2_, a phenomenon not observed in a system lacking this component. This observation indicates that an oxygen-evolving reaction is taking place; however, it does not allow the quantity or rate of O_2_ evolution to be determined. Furthermore, the use of such a significant excess of H_2_O_2_ limits the ability to extrapolate the results to conditions found in the GBM. The main limitation of the experiments presented therefore remains the lack of quantitative characterisation of oxygen production at biologically more representative substrate concentrations.

A significantly different issue regarding the oxygen balance arises in H-CeO_2_@Ce_6_@PDA@GOx. Süngü Akdogan et al. [22] They characterised the catalase-like activity of CeO_2_ by measuring the rate of H_2_O_2_ decay at initial concentrations of 0.05–1.0 mM. This range includes values similar to those determined in GBM models, but extends to approximately 15–24 times higher. For H-CeO_2_@PDA nanoparticles, a Michaelis constant (K_m_) of approximately 0.31 mM was determined, indicating a significant dependence of the reaction rate on the H_2_O_2_ concentration in the range of several tens of micromoles per litre. At concentrations of 42–69 µM, corresponding to the values described by Yin et al. [44], The Michaelis–Menten equation predicts that approximately 12–18 per cent of the maximum reaction rate will be achieved. However, this estimate relates to kinetic parameters determined in a cell-free system, whereas in GBM tissue, the course of the reaction may be further limited by substrate transport and spatial heterogeneity of the microenvironment [68,69]. Importantly, catalase-like activity was assessed on the basis of the depletion of H_2_O_2_, without simultaneous quantitative determination of the O_2_ released. Even more significant for the oxygen balance is the combination of CeO_2_ with glucose oxidase (GOx). This enzyme catalyses the oxidation of glucose to gluconolactone, consuming one molecule of O_2_ for every molecule of H_2_O_2_ produced [70]. The subsequent catalase-like decomposition of two H_2_O_2_ molecules may return one O_2_ molecule to the system. Consequently, assuming that all the H_2_O_2_ produced is directed towards the catalase-like reaction, at most half of the oxygen previously consumed by GOx is recovered. The GOx–CeO_2_ cascade itself therefore remains a process with a negative net oxygen balance, unless additional sources of O_2_ are taken into account. This balance may be even less favourable due to the co-occurrence of CeO_2_’s peroxidase-like activity, which competes with catalase-like reactions for H_2_O_2_ and leads to the formation of hydroxyl radicals (^•^OH). This ability has been demonstrated both in studies of nanostructured CeO_2_ and directly for the analysed H-CeO_2_@Ce6@PDA@GOx nanoplatform [22,71]. Although the authors confirmed the activity of individual enzymatic and catalytic modules, they did not present a quantitative balance of O_2_ production and consumption for the complete nanoplatform. This is particularly important because partial regeneration of oxygen in the cascade does not necessarily imply an increase in its total availability.

An alternative to utilising endogenous H_2_O_2_ is to introduce a dedicated oxygen-generating substrate into the nanoplatform. In the PLCNP, this function is performed by the CaO_2_ core, whose reaction with water leads to the formation of H_2_O_2_, which can then decompose to release O_2_ [48]. Chen et al. [15] They assessed how these changes depended on pH by examining the stability of the core and oxygen release over a period of up to 8 h. At pH 7, the CaO_2_ content remained virtually unchanged, whilst in an acidic environment, accelerated degradation of the material was observed. After 8 h’ incubation at pH 1, approximately 25 per cent of the initial amount of CaO_2_ remained, whereas at pH 7, approximately 90–95 per cent remained. It should be noted, however, that pH 1 differs significantly from the conditions prevailing in lysosomes, where the typical pH is around 4.5–5.0 [72].

The ability of PLCNP to release O_2_ was assessed indirectly by measuring the quenching of the luminescence of the [Ru(dpp)_3_]Cl_2_ complex by molecular oxygen. As the acidity of the environment increased, a more pronounced decrease in the probe’s signal was observed. After 8 h of incubation at pH 5.5, the luminescence intensity decreased to approximately 50% of the initial value, whilst at pH 7 it remained at around 85–90%. These results indicate increased O_2_ release under acidic conditions; however, they do not allow the absolute quantity of O_2_ or the molar yield of the CaO_2_ conversion to be determined. This is because a measurement based on luminescence quenching requires calibration of the relationship between the signal and O_2_ concentration, taking into account the properties of the environment under investigation [73]. The study did not present such a calibration for individual incubation conditions. The formation of H_2_O_2_ was additionally assessed using the Amplex Red method, which allows for its sensitive quantification but may be susceptible to interference from redox reactions and photo-oxidation of the probe itself [74]. Ultimately, the data presented confirm pH-dependent oxygen release, but do not allow us to determine what proportion of the theoretically available O_2_ was actually released, nor what the total oxygen balance of the nanoplatform was.

**Figure 2:**
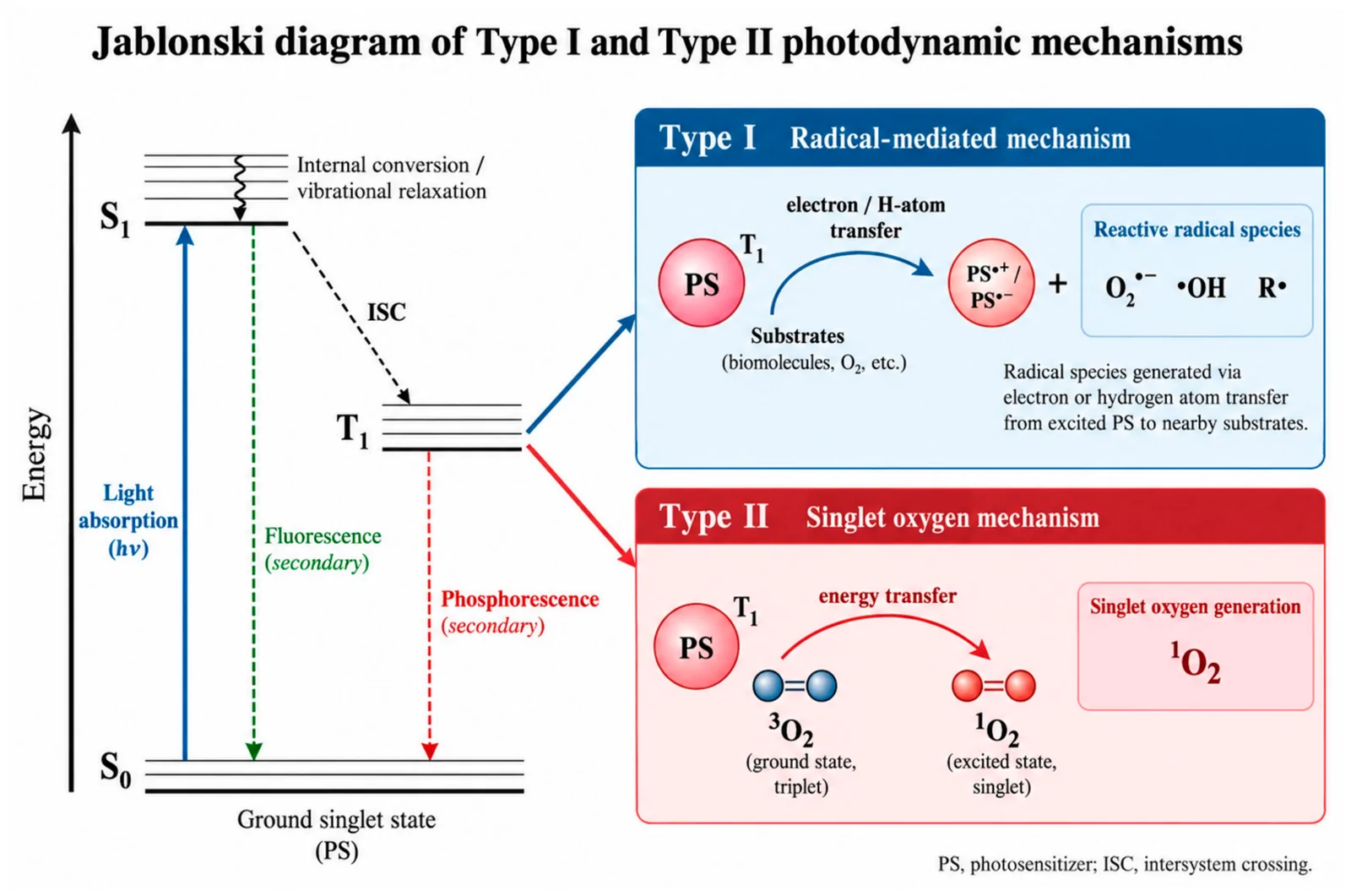
Jablonski diagram illustrating Type I and Type II photodynamic mechanisms. Following light absorption, the photosensitizer undergoes excitation from the ground singlet state (S_0_) to an excited singlet state (S_1_), followed by intersystem crossing (ISC) to the triplet state (T_1_). Type I reactions involve electron or hydrogen-atom transfer and may generate radical species, whereas Type II reactions involve energy transfer to molecular oxygen, resulting in singlet oxygen (^1^O_2_) formation. Both pathways can contribute to photodynamic activity, although their dependence on molecular oxygen differs.

Another strategy is the photocatalytic production of O_2_, as employed in FBFO@HM@aOPN and Bi_2_S_3_/Ti_3_C_2_-TPP. Zhao et al. [25] They assessed the oxygen-generating activity of the Fe_3_O_4_@BiFeO_3_ (FBFO) core using direct measurements of dissolved oxygen in solutions containing 100 µM H_2_O_2_. Upon irradiation with light at a wavelength of 660 nm and a power density of 0.5 W/cm^2^, the O_2_ concentration rose to approximately 1.6 mg/L after 30 min, whilst in a corresponding system without irradiation it reached approximately 0.5 mg/L (Figure 2). The authors attributed the increased oxygen release to the photo-induced oxidation of H_2_O_2_ by electron holes. Additional measurements carried out for the complete FBFO@HM@aOPN nanoplatform showed an increase in the concentration of dissolved O_2_ to approximately 1.3–1.4 mg/L after 30 min in the presence of H_2_O_2_, at pH 6.5 and 7.4 (Figure 2). The results obtained confirm the ability of both the core and the complete system to produce oxygen at a substrate concentration similar to that determined in GBM models [44]. However, neither the molar conversion efficiency of H_2_O_2_ to O_2_ nor the balance taking into account parallel Fenton-like reactions and the photo-induced reduction of oxygen to O_2_^•−^ was determined. The recorded changes in O_2_ concentration therefore reflect the net balance of the processes occurring in the system under study, rather than the rate of a single photocatalytic reaction.

Jiang et al. employed a different mechanism for the photocatalytic production of oxygen [24] in the Bi_2_S_3_/Ti_3_C_2_ heterostructure, which constitutes the photoactive component of the Bi_2_S_3_/Ti_3_C_2_-TPP nanoplatform. Suspensions of the material at a concentration of 100 µg/mL were deoxygenated with nitrogen, sealed with a layer of liquid paraffin, and then irradiated with light at a wavelength of 808 nm and a power density of 1 W/cm^2^. Direct measurements of dissolved oxygen showed an increase in its concentration after 20 min of approximately 4.0 mg/L for Bi_2_S_3_/Ti_3_C_2_, compared with approximately 0.7 mg/L for Bi_2_S_3_ alone. These results indicate a significantly higher oxygen-evolving activity of the heterostructure, which the authors attributed to improved separation of photogenerated charge carriers and the oxidation of water by electron holes. Unlike systems utilising H_2_O_2_, this strategy does not require the supply of peroxide as a substrate. However, despite the quantitative demonstration of an increase in O_2_ content, its origin from water was not directly confirmed, for example through isotopic labelling, the importance of which in identifying oxygen sources in photocatalytic processes was demonstrated by Montoya et al. [75]. Furthermore, the simultaneous reduction of O_2_ to O_2_^•−^ and its consumption in the oxidation of substrates by ^1O_2_ mean that the recorded increase in dissolved oxygen concentration does not allow the full balance of its production and consumption during PDT to be determined [65,76].

The analysis carried out indicates considerable variation in both the amount of O_2_ produced and the methods used to determine it. Whilst some studies employed direct measurements of dissolved oxygen, others were limited to indirect methods or qualitative observations. The most significant limitations relate to the use of H_2_O_2_ concentrations that differ from those found in the GBM, and the failure to account for concurrent oxygen consumption processes and competing redox reactions. Consequently, the demonstrated oxygen-generating activity of the nanoplatforms does not always allow their actual oxygen balance to be determined. Determining whether these processes lead to increased O_2_ availability in GBM cells and tissue requires an analysis of results obtained in biological systems.

### 4.2. Changes in Oxygen Availability in the Biological Environment

To assess the impact of nanoplatforms on GBM oxygenation, it is necessary to distinguish between direct measurements of O_2_ concentration or its partial pressure (pO_2_), indicators of cellular hypoxia, and changes in the expression of proteins involved in the hypoxic response. These methods provide complementary, but not interchangeable, information on the oxygenation status of tumour tissue [77]. Tissue pO_2_ measurements allow the local partial pressure of oxygen to be determined, whilst photoacoustic imaging, which utilises the differences in absorption between oxyhaemoglobin and deoxyhaemoglobin, primarily reflects the degree of haemoglobin oxygen saturation (sO_2_) in the vascular bed, rather than the oxygenation of tumour cells directly [78]. Conversely, HIF-1α expression serves as an indirect indicator of the cellular response, which may also be influenced by mechanisms independent of O_2_ concentration, including PI3K/AKT/mTOR signalling [77,79]. This distinction is of particular importance in GBM due to the spatial and temporal heterogeneity of oxygenation in tumour tissue [80].

The most direct assessment of tissue oxygenation was carried out by Chen et al. [15], using an oxygen microelectrode to measure pO_2_ in an orthotopic GBM model following intravenous administration of PLCNP. Measurements were taken at depths of 2 and 3 mm from the brain surface, up to 4 h after nanoparticle administration, comparing PLCNP with a control PLNP carrier lacking a CaO_2_ core, and with saline solution. The authors interpreted the results as confirming an increase in local pO_2_ following PLCNP administration; however, the data presented contain inconsistencies that make it difficult to assess them unequivocally. According to the legend for Figure S14a, the highest pO_2_ values, amounting to approximately 34–36 mmHg after 4 h, correspond to the Saline group, whilst for PLCNP the readings are approximately 2–9 mmHg, depending on the depth of measurement. Furthermore, in Panel S14b, different colour coding has been applied to these groups, which further suggests the possibility of an error in the labelling of the series in Figure S14a. Without an explanation of this discrepancy, it is not possible to unambiguously assign the presented pO_2_ values to individual groups or to quantitatively verify the claimed improvement in oxygenation. Regardless of this inconsistency, the microelectrode provides a local measurement that does not reflect the full spatial heterogeneity of GBM oxygenation [77].

Hu et al. adopted a different approach [19], evaluating Pt@PCN-222(Mn)-PEG both at the cellular level and in an orthotopic GBM model. In U87 cells, the luminescence quenching of the [Ru(dpp)_3_]Cl_2_ complex was utilised, with a reduction in signal observed following incubation with the nanoplatform, interpreted as an increase in intracellular O_2_ availability. Following irradiation, the luminescence intensity increased again, which the authors attributed to the photochemical consumption of oxygen during PDT. However, this interpretation remains qualitative, as the luminescence intensity of oxygen probes depends not only on the O_2_ concentration, but also on the amount and distribution of the indicator, photobleaching and the optical properties of the sample. More reliable quantitative measurements require appropriate calibration, and in cellular measurements, ratiometric methods or those based on luminescence lifetime are preferable [81]. The results presented therefore do not allow the absolute concentration of O_2_ or the rate of its consumption during irradiation to be determined. Changes in the probe signal were accompanied by a reduction in HIF-1α expression following application of the nanoplatform without irradiation, whilst in the Pt@PCN-222(Mn)-PEG + Laser group, a renewed increase in the level of this protein was observed. However, this increase cannot be attributed solely to the severity of hypoxia, as it has been experimentally demonstrated that PDT can induce HIF-1α activation even under normoxic conditions, amongst other things through mechanisms associated with oxidative stress and prostaglandin signalling [82]. Overall, the findings of Hu et al. are consistent with the hypothesis of a temporary increase in O_2_ availability mediated by the nanoplatform and its subsequent consumption during PDT, but do not provide quantitative confirmation of the course of these processes.

In their in vivo studies, Hu et al. [19] They used photoacoustic imaging to monitor haemoglobin saturation in an orthotopic GBM model, established by the stereotactic implantation of human U87-Luc cells into the brains of immunodeficient BALB/c nude mice (n = 5). Following intravenous administration of Pt@PCN-222(Mn)-PEG, a gradual increase in the ΔsO_2_ parameter was observed, reaching approximately 22 per cent after 12 h, followed by a decrease to approximately 8–9 per cent after 24 h. When interpreting these results, the specific nature of the model used must be taken into account. Yasui et al. [83], Using electron paramagnetic resonance imaging, they demonstrated significant differences in the oxygenation of orthotopic U87 and U251 tumours, obtaining median pO_2_ values of 29.7 and 18.2 mmHg, respectively. This suggests that the baseline level of hypoxia and the characteristics of the tumour model may significantly influence the assessment of the efficacy of oxygenation strategies. Furthermore, haemoglobin saturation reflects blood oxygenation within the tumour vasculature, rather than pO_2_ directly in the interstitial space or GBM cells [84]. Particular attention is also drawn to the time discrepancy between the maximum increase in ΔsO_2_ after 12 h and the treatment regimen used by Hu et al., in which irradiation was carried out just 2 h after administration of the nanoplatform. The measurements presented therefore do not allow us to determine whether a comparable improvement in oxygen availability was achieved during the actual PDT.

In the SOP for MN by Jiang et al. [20] They assessed the effect of the oxygen-generating module on hypoxia by measuring HIF-α expression in tumour tissues. In the subcutaneous U87-MG model, the addition of BSA–MnO_2_ to the microneedles reduced the HIF-α immunohistochemical signal by approximately 3.1-fold in the PP + BM MN group compared with the PP MN group, and by 3.0-fold in the SOP MN group compared with the PPT7 MN group. The direction of these changes was confirmed by Western blot analysis. In the orthotopic GBM model, a reduction in HIF-α expression was also observed, approximately 3.7-fold compared with the control group. These results indicate an attenuation of the molecular response associated with hypoxia; however, they do not provide direct confirmation of increased O_2_ availability in tumour tissue. The study by Vordermark and Brown [85] provides particularly compelling evidence, carried out on U87-MG cells and their xenografts. The authors demonstrated that, despite the dependence of HIF-1α expression on O_2_ concentration in vitro, only slight colocalisation of HIF-1α with pimonidazole—a recognised marker of hypoxia—was observed in tumour tissue. Furthermore, Pore et al. [86] They demonstrated in U87-MG cells that HIF-1α levels are regulated by the PI3K/Akt pathway, which influences the translation of this protein under both normoxic and hypoxic conditions. This implies that a reduction in HIF-α expression following therapy need not reflect a proportional increase in oxygen availability, but may also result from changes in signalling pathways and the biological state of the cancer cells. The study by Jiang et al. did not include direct measurements of tissue pO_2_ or an independent assessment of hypoxia using exogenous markers. A further limitation regarding interpretation is the use of a combination therapy involving PDT and induced degradation of BRD4, as this protein is also involved in the regulation of the HIF-1α pathway. Do et al. [87] demonstrated that the degradation of BRD4 led to a reduction in HIF-1α levels in cancer cells cultured under hypoxic conditions, whilst Yin et al. [88] confirmed BRD4’s involvement in the regulation of HIF1A gene transcription. Although these relationships were not directly verified in the GBM model used by Jiang et al., they suggest that the reduction in HIF-α expression may have been partly due to BRD4 degradation, rather than solely to increased O_2_ availability. Consequently, the lack of direct measurements of tissue pO_2_ limits the ability to distinguish between the two mechanisms. As a result, despite the favourable trend in HIF-α levels, the actual extent of improved oxygenation in GBM remains undetermined.

Marked changes in hypoxia markers were also described by Dash et al. [21] for rGOQD/IR820/MnO_2_/Q/CPP. A fluorescent hypoxia probe, activated under hypoxic conditions, was used in U87 cells. Following the introduction of MnO_2_, a reduction in the intensity of red fluorescence was observed compared with the rGOQD/IR820 system lacking this component. These changes were accompanied by a decrease in HIF-1α expression, as determined by Western blot analysis. In the orthotopic U87 model, the authors also assessed the immunohistochemical expression of HIF-1α following completion of therapy. The percentage of area showing positive staining was 17.5% in the rGOQD/IR820/CPP + Laser group and 0.6% in the rGOQD/IR820/MnO_2_/Q/CPP + Laser group, corresponding to a more than 29-fold reduction in the measured index. Such a significant difference confirms a marked attenuation of the molecular response associated with hypoxia; however, it does not constitute quantitative evidence of increased O_2_ availability. A significant limitation is the presence of quercetin in the complete nanoplatform, as this compound may influence the regulation of HIF-1α independently of oxygenation. Lee and Lee [89] demonstrated that quercetin inhibited the accumulation of HIF-1α in cancer cells cultured under hypoxic conditions by reducing protein synthesis. Of particular relevance to GBM, Michaud-Levesque et al. [90] demonstrated that quercetin inhibits IL-6/STAT3 signalling in U87 and T98G cells, whilst Gariboldi et al. [91] confirmed the involvement of STAT3 in the regulation of HIF-1α levels in U87-MG cells. Although it was not directly demonstrated that these mechanisms were responsible for the changes observed by Dash et al., they do suggest the possibility of O_2_-independent regulation of HIF-1α. Furthermore, the nanoplatforms under comparison differed in their simultaneous presence of MnO_2_ and quercetin, and the measurements were carried out following therapy resulting in significant damage to tumour tissue. Consequently, it is not possible to unequivocally attribute the reduction in HIF-1α expression solely to the oxygen-generating activity of MnO_2_, nor to determine the actual extent of improved oxygenation in GBM.

In the case of FBFO@HM@aOPN, Zhao et al. [25] They analysed changes in hypoxia within three-dimensional spheroids formed from CT2A cells and M2-phenotype macrophages. The fluorescent indicator ‘Hypoxia Green Reagent’ was used to assess hypoxia; its signal increases under conditions of reduced O_2_ availability. The usefulness of green hypoxia probes has also previously been demonstrated in three-dimensional GBM models, using confocal imaging and quantitative fluorescence analysis across individual layers of the spheroids [92]. Zhao et al. observed the weakest fluorescence in spheroids treated with FBFO@HM@aOPN, including in their central regions, whilst the signal remained significantly stronger in the PBS and FBFO groups. These results are consistent with hypoxia, although the method used provides only an indirect assessment of oxygen availability. The authors did not present a calibration of the fluorescence signal against O_2_ concentration, nor did they provide a quantitative analysis of its spatial distribution. An additional limitation is the possibility of uneven penetration of the fluorescent probes into the interior of the spheroids, the significance of which was demonstrated experimentally by Zhang et al. [93]. The study by Zhao et al. did not provide an independent assessment of the distribution of the tracer used within the individual layers of the spheroids. Furthermore, the authors demonstrated that hybrid surface modification of the nanoparticles increased the penetration of FBFO into the interior of the spheroids compared with an uncoated core. The observed differences in the hypoxia signal may therefore reflect both the oxygen-generating activity of the material and the increased availability of nanoparticles in the deeper layers of the model. Although the results provide evidence supporting a reduction in hypoxia, they do not allow for the determination of the actual increase in O_2_ concentration, nor do they allow the effects of the photocatalytic properties and the altered distribution of the nanoplatform to be distinguished.

An indirect assessment of the effect of the Bi_2_S_3_/Ti_3_C_2_-TPP nanoplatform on hypoxia was carried out by Jiang et al. [24]. In U251 cells incubated under hypoxic conditions, the Image-iT Red fluorescent indicator was used, the signal from which increases when O_2_ availability is reduced. Following irradiation of the nanoplatform with light at a wavelength of 808 nm and a power density of 1 W/cm^2^ for 10 min, a reduction in fluorescence intensity was observed compared with a control system without irradiation. This result is consistent with an increase in oxygen availability following the photocatalytic activation of the nanoplatform; however, the method used provides only an indirect assessment of its concentration. The authors did not present a calibration of the fluorescence signal against O_2_ nor direct measurements of cellular oxygenation. Furthermore, oxygen availability at the cell surface depends not only on its concentration in the culture medium, but also on its transport through the medium and metabolic consumption [94]. The observed changes in fluorescence therefore do not allow for a quantitative assessment of the improvement in oxygenation or the rate of O_2_ production in the cellular environment.

In the in vivo studies, a subcutaneous U251 xenograft model was used in immunodeficient BALB/c nude mice, with treatment commencing when tumour volume was approximately 80 mm^3^. The most pronounced reduction in HIF-1α expression was observed following intravenous administration of Bi_2_S_3_/Ti_3_C_2_-TPP and irradiation with 808 nm light. Although this result is consistent with the proposed mechanism of hypoxia mitigation, HIF-1α expression is not a direct measure of tissue pO_2_, and changes in its levels may also result from processes independent of oxygenation [85]. Another significant limitation is the use of a subcutaneous model, whose hypoxia profile may differ significantly from the conditions found in intracranial GBM. Bürgi et al. [95], When comparing subcutaneous and orthotopic GL261 tumours, they demonstrated stronger activation of the HIF pathway and more extensive areas of hypoxia in the subcutaneous model. The maximum induction of HIF-dependent reporter activity was 7.6- and 3.2-fold the baseline value, respectively, with these maxima occurring at different stages of tumour growth. The authors attributed the observed differences, amongst other things, to differences in vascularisation and tumour size. The significance of tumour size was also confirmed by Wang et al. [96], showing an increase in hypoxia as U251 tumours grew, both in subcutaneous and intracranial locations. These results indicate that the location and growth conditions of the tumour significantly influence the characteristics of hypoxia; therefore, the changes in HIF-1α expression observed by Jiang et al. cannot be directly extrapolated to orthotopic GBM.

An additional limitation on interpretation is the co-occurrence of photodynamic and photothermal effects. Jiang et al. demonstrated an increase in tumour surface temperature from 31.2 to 51.9 °C after 5 min of irradiation, as well as extensive necrotic changes in the Bi_2_S_3_/Ti_3_C_2_-TPP + Laser group. The significance of the thermal effect for the interpretation of HIF-1α is confirmed by the studies of Xu et al. [97], who, in the 4T1 breast cancer model, demonstrated that PTT leads to temporally and spatially varied changes in HIF-1α expression, including an initial decrease followed by a subsequent increase, occurring in conjunction with changes in perfusion and damage to tumour tissue. Moon et al., on the other hand, ref. [98] demonstrated that hyperthermia alone can increase the stabilisation of HIF-1α via mechanisms dependent on ROS and the ERK pathway, even under normoxic conditions. Although these relationships have been described in other cancer models, they demonstrate that the HIF-1α response to heating does not necessarily directly reflect changes in O_2_ availability. Consequently, the reduction in HIF-1α expression observed by Jiang et al. cannot be unequivocally attributed to photocatalytic oxygen production, as it may also have been influenced by the effects of combination therapy. Despite the consistent direction of changes in hypoxia markers in cellular and in vivo studies, the lack of direct measurements of O_2_ or tissue pO_2_, as well as appropriate controls to distinguish between photocatalytic and photothermal effects, precludes a quantitative assessment of the actual improvement in GBM oxygenation.

In the case of H-CeO_2_@Ce6@PDA@GOx, Süngü Akdogan et al. [22] They did not provide direct evidence of increased oxygen availability in a biological environment. The studies were conducted exclusively in vitro, using two-dimensional cultures of human T98G glioma cells, whilst the enzymatic and catalase-like activity of the nanoplatform was assessed in separate cell-free systems. The presence of active glucose oxidase (GOx) was confirmed on the basis of the rate of glucose consumption, with a maximum reaction rate of 7.61 mM/min determined and an estimated retention of approximately 85.7 per cent of free enzyme activity. It should be noted, however, that the reference value for free GOx was calculated on the basis of catalogue data, rather than parallel measurements under identical conditions. In turn, the catalase-like activity of CeO_2_ was assessed by measuring the depletion of H_2_O_2_, showing its complete decomposition within 60 min for H-CeO_2_ and H-CeO_2_@PDA. These measurements confirm the ability of individual components to catalyse the relevant reactions, but do not allow the amount of O_2_ actually produced in the complete nanoplatform to be determined. Furthermore, enzymatic activity assays were carried out on materials not containing Ce^6^, due to interference from the photosensitiser in colorimetric measurements, and GOx and catalase-like activities were characterised at different temperatures, 37 and 22 °C respectively. This limits the possibility of quantitatively extrapolating the obtained parameters to the simultaneous course of the reaction in the complete system. The importance of directly determining the oxygen product and the influence of measurement conditions on the assessment of nanozyme activity was demonstrated by Mirra et al. [64], by comparing, amongst other things, the rate of H_2_O_2_ decomposition and O_2_ evolution by CeO_2_ nanoparticles.

Particularly important caveats arise from the presence of GOx, whose activity is linked to oxygen consumption. Baumann et al. [99] They demonstrated experimentally that the GOx–catalase cascade, despite the partial regeneration of O_2_ in the catalase reaction, leads to its rapid depletion in solutions and cell culture media. Under the conditions employed by the authors, the O_2_ concentration fell to below 1 per cent of air saturation within approximately 1 min and approached zero after 3 min; the biological effects of the resulting hypoxia were also confirmed in U251 glioma cells. Although the system using natural catalase employed by Baumann et al. differs from the CeO_2_-containing nanoplatform, these results demonstrate that the co-existence of GOx and catalase-like activity is not a sufficient argument for increased oxygen availability.

In the study by Süngü Akdogan et al., neither the concentration of dissolved nor intracellular O_2_ was measured, the expression of HIF-1α was not assessed, nor were independent markers of hypoxia employed. Nor was a comparison presented of changes in T98G cell oxygenation under controlled normoxic and hypoxic conditions. This is of particular significance given the limitations of two-dimensional cell cultures, which do not replicate the spatial oxygen gradients characteristic of GBM. Hubert et al. [100] They demonstrated that three-dimensional organoids derived from human gliomas develop distinctly differentiated oxygenation zones, including central hypoxic regions, which are not reproduced in standard monolayer cultures. Furthermore, the actual availability of O_2_ at the cell surface depends on its transport through the medium and the rate at which it is consumed; therefore, it cannot be determined solely on the basis of the composition of the culture atmosphere [94]. Consequently, the results presented document the activity of the components of the GOx–CeO_2_ cascade, but do not determine whether its action increases or decreases the availability of O_2_ in GBM cells. The claimed ability of the nanoplatform to reduce hypoxia therefore remains unconfirmed in the biological model used.

The analysis carried out indicates that the evidence for increased O_2_ availability in the biological environment remains inconsistent and is based mainly on indirect indicators of hypoxia. Direct measurements of oxygenation have been carried out in only a few studies, and the interpretation of the results is limited by the non-specificity of the markers used, the influence of additional therapeutic mechanisms, and differences between GBM models. Consequently, the observed changes do not always allow for unequivocal confirmation of an actual improvement in tumour oxygenation, particularly during PDT.

### 4.3. Maintenance of Photochemical Activity During Hypoxia

Assessing the ability of nanoplatforms to maintain photodynamic activity under hypoxic conditions requires a distinction to be made between the generation of reactive oxygen species (ROS), the maintenance of photocytotoxicity and the ultimate therapeutic effect, as these parameters relate to different stages of PDT and cannot be regarded as equivalent measures of its efficacy [65,101]. The most reliable studies are those in which PDT activity has been compared at controlled O_2_ concentrations, taking into account appropriate control groups and the specific characteristics of the photochemical reactions [102]. The time taken for cells to adapt to hypoxia and the maintenance of aerobic conditions during irradiation are also of significant importance, as the actual availability of O_2_ in the cellular environment depends on its transport and metabolic utilisation [94]. Equally important is the specificity of the methods used to measure ROS, as their limitations may lead to the misidentification of photochemical reaction products [103]. Merely demonstrating cytotoxicity in an atmosphere with reduced O_2_ content is therefore not equivalent to quantitative confirmation of the efficacy of individual photodynamic reactions, particularly in nanoplatforms that combine PDT with other therapeutic mechanisms.

Zhang et al. carried out relatively well-controlled experiments [28] for the LTPA nanoplatform. In U87 cells, ROS generation and photocytotoxicity were compared under normoxic (21% O_2_) and hypoxic (2% O_2_) conditions, following an eight-h adaptation to reduced oxygen availability. The formation of ^^1^O_2_, O_2_^•−^ and ^•^OH was assessed using SOSG, DHE and HPF probes, respectively. Under both oxygen conditions, similar fluorescence intensities attributed to the generation of O_2_^•−^ and ^•^OH were observed, whilst the SOSG signal remained low. These results were consistent with the proposed predominance of Type I reactions. Furthermore, the relative cell viability assessed by the CCK-8 assay remained comparable at 21% and 2% O_2_. However, this result must be interpreted with caution, as the CCK-8 assay, which is based on the reduction of the tetrazolium salt WST-8, reflects the metabolic activity of cells, which may not necessarily correspond to their actual survival rate, particularly under conditions of hypoxia and oxidative stress induced by PDT. An additional limitation is the potential for nanomaterials to interfere with the assay, as demonstrated, amongst other things, for CuO nanoparticles, although this does not constitute evidence of analogous interference in the case of LTPA [104,105]. However, the reliability of the results obtained is reinforced by independent Calcein-AM/PI and Annexin V-FITC/PI assays, which confirmed cell damage and death following irradiation in both normoxia and hypoxia.

It was particularly important to conduct additional experiments distinguishing between the contributions of PDT and PTT. After limiting the photothermal effect by cooling the samples to 4 °C, the relative viability assessed by the CCK-8 assay was approximately 60% in both normoxia and hypoxia, whilst combination therapy reduced it to approximately 30%. These results indicate that the activity of the PDT component is maintained at 2% O_2_ even after the contribution of PTT has been reduced; however, they are based solely on metabolic measurements, without independent verification of cell death under these specific experimental conditions.

These results provide direct evidence that the photocytotoxicity of PDT is maintained at 2% O_2_, even after the contribution of PTT has been reduced. However, interpretation is complicated by the method used to distinguish between mechanisms, as a reduction in temperature affects cellular processes, whilst N-acetylcysteine (NAC), used to limit oxidative effects, may modify intracellular redox pathways independently of the direct quenching of ROS [106,107]. Furthermore, it was not specified whether an atmosphere containing 2% O_2_ was maintained during cooling, which limits the possibility of unequivocally attributing the result to strictly controlled hypoxia conditions.

A further limitation concerns the specificity of the DHE probe, whose fluorescence does not allow for the unambiguous quantification of O_2_^•−^ without separating the oxidation products [108]. Despite these caveats, the results provide relatively strong evidence for the maintenance of PDT activity under hypoxic conditions at the cellular level. However, the efficiency of ROS generation in areas with varying oxygenation levels has not been directly verified in an orthotopic GBM model.

Equally important data on the maintenance of PDT activity under hypoxic conditions were provided by Caldas et al. [30], by investigating the RhB-MNPs nanoplatform in U87-MG cells. A controlled atmosphere containing 1% O_2_ was also maintained during irradiation, which reduced the risk of unintended reoxygenation of the cells associated with transferring the culture to normoxic conditions [109]. Under hypoxic conditions, the relative viability of U87-MG cells, as assessed by the MTT assay, was 49 ± 8% following PDT and was similar to that observed at 21% O_2_. This result indicates that the nanoplatform retains its phototoxic activity under hypoxic conditions; however, the MTT assay, which is based on cellular metabolic activity, does not allow for an unambiguous determination of the actual survival rate of the cells following PDT [110]. Furthermore, the authors found no significant phototoxicity of the melanin nanoparticles themselves under the irradiation parameters used, which limits the possibility of attributing the observed phototoxic effect to the properties of the carrier. The results therefore indicate that RhB-MNPs retain their biological activity despite limited oxygen availability.

Additional experiments with reactive oxygen species (ROS) scavengers suggested the involvement of both Type I and Type II PDT mechanisms. However, the interpretation of these results is limited by the non-specificity of the DCFH-DA probe used, which does not allow for the unambiguous identification of individual ROS [103]. As no mechanistic analysis was carried out under hypoxic conditions, the results obtained do not allow us to determine which of the mechanisms was responsible for the preservation of photocytotoxicity at 1% O_2_. Furthermore, the interpretation of the results is limited by the 90-min period of exposure to hypoxia prior to irradiation, which does not fully replicate the chronic adaptive changes characteristic of GBM. Nor was the O_2_ concentration in the immediate vicinity of the cells measured directly; this may not correspond to its concentration in the culture atmosphere [111]. The lack of in vivo validation further limits the possibility of extrapolating the results obtained to the complex tumour microenvironment. The study therefore provides direct evidence of the photocytotoxicity of RhB-MNPs in the in vitro hypoxia model used, without, however, determining the mechanism responsible for this effect.

Chen et al. evaluated a different strategy for reducing the dependence of PDT on oxygen availability [15], using a PLCNP nanoplatform containing a CaO_2_ core capable of generating O_2_. In an orthotopic GBM model, a significant increase in local oxygen partial pressure was observed following PLCNP administration, confirming the nanoplatform’s ability to improve tumour tissue oxygenation. However, in glioma-initiating cells (GICs), maintained at 1% O_2_ for 24 h prior to PDT and during subsequent stages of the experiment, the use of PLCNP + PDT led only to a statistically non-significant reduction in relative viability, as assessed by the CCK-8 assay, compared with PLNP + PDT. A comparison with a carrier lacking a CaO_2_ core therefore did not confirm a significant improvement in photocytotoxicity under the hypoxic conditions used. However, the interpretation of this result is limited by the lack of independent verification of cell death following PDT at 1% O_2_, as the metabolic assay used does not allow for the distinction between actual loss of viability and changes in redox activity. Furthermore, potential interference of the nanoplatform’s components with the CCK-8 assay was not assessed; such interference cannot be ruled out, particularly in a system containing a reactive CaO_2_ core.

A comparison with a carrier lacking a CaO_2_ core therefore did not confirm a significant improvement in photocytotoxicity under the hypoxic conditions used.

Separate assays using DCFH-DA showed an increase in the oxidative signal following the application of PLCNP, whilst measurements using Amplex Red indicated increased levels of H_2_O_2_. As these experiments did not include an assessment of ROS generation during irradiation under controlled hypoxia, they do not allow us to determine whether the presence of the CaO_2_ core increased the efficiency of photodynamic reactions at 1% O_2_. Nor was the generation of ^1^O_2_ during PDT under these conditions quantified. Although the therapeutic efficacy of PLCNP was confirmed in an orthotopic GBM model following tumour resection, the efficiency of photodynamic reactions in hypoxic tissue during irradiation was not directly assessed. The results therefore confirm the ability of PLCNP to increase local O_2_ availability; however, evidence that this mechanism translates into improved PDT efficacy under hypoxic conditions remains limited.

Significant evidence of the maintenance of PDT activity under conditions of limited oxygen availability was provided by Zhang et al. [27], by investigating the TTTMN NPs-Ang2 nanoplatform in GL261 cells. Hypoxia was induced using a sealed chamber and a chemical oxygen-scavenging system, which, according to the manufacturer’s specifications, provides an atmosphere containing less than 1% O_2_. Under these conditions, oxidative activity was shown to be maintained following irradiation, as confirmed by the relative fluorescence intensities of the DCFH-DA and DHR123 probes, which were comparable to those obtained under normoxia. Similarly, relative cell viability, as assessed by the CCK-8 assay, remained comparable under both oxygen conditions. Although this result does not constitute a direct measurement of cell death, its consistency with independent measurements of oxidative signals strengthens the evidence for the preservation of PDT activity under hypoxic conditions. The authors also confirmed the induction of cell death using Calcein-AM/PI and Annexin V-FITC/PI staining; however, these assays were not performed under separately documented hypoxic conditions and therefore do not allow for independent confirmation of comparable photocytotoxicity under conditions of limited O_2_ availability.

Although the authors quantitatively compared the relative fluorescence signals under normoxia and hypoxia, the non-specificity of both probes does not allow for an unambiguous quantification of individual ROS. In particular, the oxidation of DHR123 is not specific to O_2_^•−^ and may involve other oxidising agents and peroxidase systems [103,112]. Furthermore, the authors did not measure the actual concentration of O_2_ in the chamber atmosphere or in the culture medium, which limits the ability to establish a quantitative link between the observed activity and a specific level of hypoxia. The therapeutic efficacy of TTTMN NPs-Ang2 was also confirmed in an orthotopic GBM model using fibre-optic radiation delivery; however, ROS generation in hypoxic tumour regions was not directly assessed. The study therefore provides relatively strong evidence of the preservation of photodynamic activity under in vitro hypoxia, although the lack of precise control over O_2_ concentration and selective ROS assays limits the quantitative assessment of this effect. Furthermore, independent ESR measurements demonstrated the generation of ^•^OH and O_2_^•−^ radicals, confirming the involvement of Type I reactions, although without direct verification in cells maintained under hypoxic conditions.

Another group comprises nanoplatforms, which have been shown to retain their biological activity following irradiation under conditions of reduced O_2_ availability; however, limitations in the methods used and the co-occurrence of additional therapeutic mechanisms make it difficult to assess the contribution of PDT alone. An example is JSK@PEG-IR820, described by Zou et al. [29]. In U87-MG cells, under an atmosphere containing 2% O_2_, a concentration-dependent reduction in relative viability, as assessed by the MTT assay, was observed, down to approximately 29% at the highest concentration of the nanoplatform tested. This result indicates that the system retains its biological activity following irradiation under hypoxic conditions; however, its interpretation is limited by the method used. The MTT assay reflects the metabolic activity of cells, which may not necessarily correspond to their actual survival, particularly following PDT. Rodríguez et al. demonstrated discrepancies between MTT results and those of the clonogenic assay, which assesses the ability of cells to proliferate further following irradiation [110]. As Zou et al. did not carry out an independent verification of cell death at 2% O_2_, the result obtained does not constitute conclusive evidence of sustained photocytotoxicity. Furthermore, the lack of a direct comparison with normoxia and appropriate controls without irradiation under hypoxic conditions limits the ability to assess the actual impact of hypoxia on the efficacy of PDT.

In separate fluorescence experiments, the authors detected signals attributed to O_2_^•−^, NO and ONOO^−^, consistent with the proposed interaction between Type I photodynamic reactions and the release of NO from the JSK component. However, these measurements were not documented at 2% O_2_, and therefore do not allow for a direct assessment of ROS generation efficiency under hypoxic conditions. Furthermore, the formation of ONOO^−^ in the reaction of NO with O_2_^•−^ may exacerbate cellular damage [58], However, based on the results presented, it is not possible to quantitatively distinguish the contribution of this sary mechanism of cytotoxicity from the direct effect of PDT. Although the therapeutic efficacy of JSK@PEG-IR820 was also demonstrated in the subcutaneous U87-MG model, ROS generation in hypoxic tumour regions was not assessed. The study therefore indicates that the nanoplatform retains its biological activity following irradiation under hypoxic conditions; however, it does not provide sufficient evidence to confirm sustained photocytotoxicity or to quantitatively determine the contribution of PDT alone to the observed effect.

Similar limitations are present in the Bi_2_S_3_/Ti_3_C_2_-TPP studies conducted by Jiang et al. [24]. In U251 cells, the effect of the nanoplatform was assessed under normoxic and hypoxic conditions, for which the culture protocol specified an atmosphere containing 1% O_2_. In a direct comparison carried out in air and nitrogen atmospheres, the relative viability assessed by the MTT assay following irradiation was approximately 10% and 12%, respectively. The results indicate that the nanoplatform retains strong biological activity despite limited oxygen availability. However, their interpretation is limited by the use of the MTT assay, which reflects the metabolic activity of the cells rather than their survival directly [113]. Furthermore, nanomaterials may interfere with MTT assays, and the occurrence of such interference has not been verified for the nanoplatform under investigation [114]. As the authors did not specify the actual O_2_ concentration during the comparison between air and nitrogen atmospheres, it is not possible to unequivocally attribute the results obtained to conditions of exactly 1% O_2_.

Furthermore, in hypoxic cells, a reduction in the Image-iT probe signal was observed following the application of Bi_2_S_3_/Ti_3_C_2_-TPP and irradiation, which provides indirect evidence of improved intracellular oxygen availability. In separate experiments, an increase in the DCFH-DA signal was demonstrated following irradiation, whilst ESR measurements in model systems indicated the generation of ^1^O_2_, O_2_^•−^ and ^•^OH. However, no selective, quantitative assessment of the generation of individual ROS was carried out in cells maintained at 1% O_2_.

A key aspect of the study was the attempt to distinguish the contributions of PDT and PTT by comparing MTT assay results following irradiation at 37 and 4 °C. On this basis, the authors estimated the contribution of PDT to the total cytotoxic effect at approximately 69.2 per cent. However, this result should be regarded as an approximation, as a reduction in temperature may affect metabolic activity and mitochondrial function independently of the reduction in the photothermal effect [115]. Furthermore, it has not been demonstrated that this experiment was carried out under strictly controlled O_2_ concentrations. Although the therapeutic efficacy of the nanoplatform has also been demonstrated in the subcutaneous U251 model, the generation of ROS in hypoxic tumour regions has not been directly assessed. The study therefore provides evidence that Bi_2_S_3_/Ti_3_C_2_-TPP retains its biological activity following irradiation under conditions of limited oxygen availability; however, it does not allow for unequivocal confirmation of permanent cell death nor does it determine the contribution of PDT alone to the observed effect under hypoxic conditions.

Zhao et al. obtained less conclusive results [25], when investigating the FBFO@HM@aOPN nanoplatform in CT2A cells. In experiments comparing normoxia and hypoxia, the relative viability, as assessed by the CCK-8 assay following application of the nanoplatform and irradiation, was approximately 17 per cent and 40 per cent, respectively). The results indicate that the biological activity of the system is maintained under conditions of limited oxygen availability; however, its efficacy was markedly lower than under normoxia. The authors did not specify the exact O_2_ concentration nor did they document the conditions under which it was maintained during irradiation, which makes it impossible to correlate the observed activity with a specific level of hypoxia. Interpretation is further limited by the use of the CCK-8 assay, which reflects metabolic activity, without independent confirmation of cell death under the compared oxygen conditions. Although the authors also carried out an analysis of apoptosis using the Annexin V-FITC/PI method, they did not document its performance under controlled hypoxia.

In separate experiments, the ability of the nanoplatform to generate ROS radicals, including ^•^OH and O_2_^•−^, was demonstrated using ESR and reactions with chemical probes. However, no direct quantitative comparison of their generation in cells under varying O_2_ availability was carried out. Furthermore, the interpretation of the observed effects is complicated by the Fenton-like activity of the core, which may contribute to oxidative damage independently of the primary photodynamic reactions. Although the therapeutic efficacy of FBFO@HM@aOPN has also been confirmed in an immunocompetent, orthotopic CT2A model, the generation of ROS in hypoxic tumour regions during irradiation was not directly assessed. The study therefore indicates that the cytotoxic activity of the entire nanoplatform is maintained in the hypoxia model used; however, it does not allow for unequivocal confirmation of the efficacy of PDT alone, nor does it determine its contribution to the observed effect.

In the case of SOP MN, Jiang et al. [20] assessed the effect of BSA–MnO_2_ on the photodynamic activity of the PPT7 module in U87-MG cells. The experiments were conducted under hypoxic conditions (1% O_2_, 5% CO_2_, 94% N_2_), following a 24-h adaptation period for the cells to reduced oxygen availability. Upon combining PPT7 with BSA–MnO_2_, an approximately two-fold increase in the relative fluorescence intensity attributed to ^1^O_2_ generation was observed. However, the interpretation of this result is limited by inconsistencies in the documentation of the probe used: the main text refers to DCFH-DA, which is non-specific for ^1^O_2_, whilst the supplementary SOSG methodology, the response of which may also be subject to photochemical interference [103,116]. At the same time, the IC_50_ value, determined on the basis of the CCK-8 assay after 72 h, decreased from 0.18 µg/mL for PPT7 + PDT to 0.09 µg/mL for PPT7 + BSA–MnO_2_ + PDT. Although this assay reflects metabolic activity rather than cell survival directly, an independent indicator of apoptotic pathway activation was the increase in cleaved caspase-3 levels, demonstrated by Western blot analysis under hypoxic conditions. This marker indicates the activation of the executor mechanisms of apoptosis; however, on its own, it does not confirm irreversible cell death [117].

Interpreting this increased therapeutic efficacy is complicated by the interaction between PDT and the activation of the PROTAC prodrug, as the ^1^O_2_ generated is involved both in direct cell damage and in the release of ARV-771, leading to the degradation of BRD4. The significance of this mechanism in GBM is confirmed by studies indicating that BRD4 is involved in the regulation of glioma cell proliferation [118]. The authors demonstrated BRD4 degradation following irradiation even under hypoxic conditions, whilst in an orthotopic GBM model they observed a 2.8-fold reduction in BRD4 levels and a 3.7-fold decrease in HIF-α expression. This latter finding is particularly significant in the context of GBM’s adaptation to hypoxia, as HIF-1α signalling promotes the maintenance of the stem-cell-like phenotype of glioma cells [119]. As noted earlier, however, a reduction in HIF-α expression does not constitute direct evidence of improved tumour oxygenation, as the regulation of this protein may also occur independently of O_2_ availability [120].

The results obtained therefore indicate a beneficial effect of BSA–MnO_2_ on the photodynamic activity of PPT7 and the efficacy of the entire system under hypoxic conditions; however, no direct measurement of the O_2_ concentration in the cellular environment during irradiation was presented, nor was there a quantitative assessment of ^1^O_2_ generation in hypoxic tumour regions. The study thus provides significant evidence of the system’s continued activity under conditions of limited oxygen availability, but does not allow for a quantitative determination of the extent to which BSA–MnO_2_ enhances the efficiency of the photodynamic reactions themselves under hypoxic conditions.

In some studies on nanoplatforms designed to counteract GBM hypoxia, an increase in O_2_ availability, ROS generation following irradiation and improved therapeutic efficacy have been demonstrated; however, there has been no direct verification of the maintenance of PDT activity under conditions of controlled oxygen deprivation. Hu et al. [19] demonstrated changes in oxygenation indices in a cellular model of CoCl_2_-induced hypoxia and an increase in oxygen saturation in an orthotopic GBM model, but did not compare ROS generation efficiency and photocytotoxicity at defined O_2_ levels. In contrast, Dash et al. [21] observed a reduction in the cellular signal of the hypoxia marker and an increase in the relative fluorescence intensity of DCFH-DA following the introduction of MnO_2_, although they did not directly compare photodynamic activity under controlled normoxia and hypoxia. Similarly, Li et al. [23] and Süngü Akdogan et al. [22] confirmed the ability of the developed nanoplatforms to generate ROS and induce cytotoxic effects following irradiation, but did not determine how these processes depend on the availability of O_2_. These results support the proposed mechanisms of action of the nanoplatforms, but do not constitute direct evidence of the maintenance of PDT efficacy under hypoxic conditions.

A comprehensive analysis indicates that the strongest evidence for the preservation of PDT activity under hypoxic conditions comes from studies involving controlled hypoxic conditions, appropriate control groups and a parallel assessment of ROS generation and photocytotoxicity. The interpretation of the remaining results is mainly limited by the lack of precise control over O_2_ concentration, the non-specificity of ROS probes, and the co-occurrence of additional therapeutic mechanisms. Although PDT activity has been shown to be maintained under hypoxic conditions at the cellular level for some nanoplatforms, and their efficacy has also been confirmed in orthotopic GBM models, there is still a lack of direct, quantitative assessment of the efficiency of photodynamic reactions in hypoxic tumour regions (Table 1).

## 5. Therapeutic Efficacy

### 5.1. In Vitro Antitumor Efficacy

Research into photodynamic nanoplatforms designed for the treatment of glioblastoma has demonstrated varying degrees of inhibition of proliferation and induction of cancer cell death. Efficacy was assessed primarily on the basis of relative cell viability, IC_50_ values, staining of live and dead cells, analysis of apoptosis, and colony-forming capacity. In the case of multimodal nanoplatforms, comparisons of relevant therapeutic variants also allowed for an assessment of the benefits arising from the combination of PDT with other treatment methods.

Zhang et al. [28] They demonstrated strong LTPA photocytotoxicity against U87 cells, confirmed by the CCK-8 assay and Calcein-AM/PI staining. The use of combined PDT/PTT led to a reduction in relative cell viability to approximately 30 per cent, whilst after limiting the photothermal component, it was approximately 60 per cent. Additional Annexin V-FITC/PI analysis confirmed the induction of apoptosis, whilst lipid peroxidation assays also indicated the activation of ferroptotic processes.

In the case of RhB-MNPs, Caldas et al. [30] They demonstrated a significant reduction in the relative viability of U87-MG cells following irradiation. In the MTT assay, a value of 49 ± 8 per cent was obtained under hypoxic conditions, whilst a similar effect was also observed under normoxic conditions. The mere presence of melanin nanoparticles did not cause a comparable phototoxic effect, whereas combining them with rhodamine B increased the efficacy of photo-induced cell damage.

In the TTTMN NPs-Ang2 studies, Zhang et al. [27] showed a marked reduction in the relative viability of GL261 cells following irradiation. Additional Calcein-AM/PI and Annexin V-FITC/PI staining confirmed the induction of cell death. A stronger effect was achieved by delivering radiation directly to the cells via an optical fibre, compared with irradiation through a layer of tissue.

Chen et al. [15] demonstrated increased efficacy of PLCNP in glioblastoma initiating cells (GICs) compared with the control PLNP carrier. Following photodynamic activation, PLCNP caused a greater reduction in relative cell viability, as confirmed in cytotoxicity experiments. The authors also assessed the response of GICs to various treatment regimens, demonstrating the beneficial effect of using the complete nanoplatform.

Jiang et al. achieved a significant improvement in anticancer activity [20] for SOP MN. In U87-MG cells, the IC_50_ value determined by the CCK-8 assay decreased from 0.18 µg/mL for PPT7 + PDT to 0.09 µg/mL following the combination of PPT7 with BSA–MnO_2_. This increased anti-tumour activity was accompanied by a rise in the level of cleaved caspase-3, confirming the activation of the apoptotic pathway. In the case of the complete therapeutic regimen, an additional effect was achieved through the combination of PDT with BRD4 degradation.

Zou et al. [29] demonstrated increased cytotoxicity of JSK@PEG-IR820 against U87-MG cells compared with the individual components of the system. The IC_50_ value of the complete nanoplatform following irradiation was 1.4 µg/mL. An Annexin V-FITC/PI flow cytometric analysis revealed apoptosis in 43.3% of cells following treatment with JSK@PEG-IR820 and irradiation, compared with 31.1% in the JSK group and 20.9% for PEG-IR820 with irradiation. The results of Calcein-AM/PI staining were consistent with the observed increase in cytotoxicity.

A strong multimodal effect was also described by Jiang et al. [24] for Bi_2_S_3_/Ti_3_C_2_-TPP. In U251 cells, the relative viability following irradiation decreased to 8.9 per cent, which was the lowest value amongst the compared nanomaterial variants. An Annexin V-FITC/PI analysis revealed a combined percentage of apoptotic and necrotic cells of 33.73 per cent, compared with 23.43 per cent for Bi_2_S_3_/Ti_3_C_2_ without TPP modification. Furthermore, the colony formation assay demonstrated a significant reduction in the cells’ proliferative capacity following treatment.

In the case of Fe_3_O_4_@PDA@Ru, Li et al. [23] demonstrated concentration-dependent anticancer activity against U87-MG. Following combined PDT and PTT activation, the IC_50_ value was 0.0153 mg/mL, or 15.3 µg/mL. The MTT results were supplemented by Calcein-AM/PI staining and flow cytometric analysis, which confirmed an increase in cell death following irradiation.

Süngü Akdogan et al. [22] demonstrated high efficacy of the combination therapy using H-CeO_2_@Ce6@PDA@GOx against T98G cells. At nanoplatform concentrations of 1–2 mg/mL, the relative viability following the simultaneous application of ST, CDT, PDT and PTT ranged from 10.6% to 13.1%. The MTT results were consistent with the AO/PI staining, indicating a significant proportion of dead cells following the combination therapy.

Hu et al. [19] confirmed the efficacy of Pt@PCN-222(Mn)-PEG against U87 cells, observing a concentration-dependent reduction in relative viability in the CCK-8 assay. Light irradiation further enhanced the anticancer activity compared with the nanoplatform without light activation. This effect was confirmed by flow cytometry using Annexin V-FITC/PI and Calcein-AM/PI staining.

In turn, Dash et al. [21] demonstrated increased cytotoxicity of the complete rGOQD/IR820/MnO_2_/Q/CPP nanoplatform against U87 cells compared with variants lacking individual therapeutic components. The MTT results indicated a significant reduction in relative viability following irradiation, whilst flow cytometric analysis confirmed increased levels of apoptosis and necrosis.

Zhao et al. [25] demonstrated significant photocytotoxicity of FBFO@HM@aOPN against CT2A cells, as confirmed by the CCK-8 assay and flow cytometry. In addition, enhanced immunogenic cell death was observed, as evidenced by increased calreticulin exposure and the release of ATP and HMGB1. Experiments with macrophages also demonstrated increased phagocytosis of tumour cells, representing an additional anti-tumour effect of the nanoplatform.

### 5.2. In Vivo Antitumor Efficacy

In in vivo studies, therapeutic efficacy was assessed primarily on the basis of changes in tumour volume, bioluminescent imaging, histopathological analyses, tumour recurrence and animal survival. In the majority of experiments, the combination of the nanoplatform with radiation therapy led to greater inhibition of GBM growth than in the corresponding control groups.

Some of the most striking results regarding prolonged survival were reported by Chen et al. [15] for PLCNP. In an orthotopic GBM model derived from GICs, the combination of surgical resection with PLCNP and PDT resulted in the smallest volume of residual tumour tissue compared with the other treatment options. The authors also demonstrated a tumour elimination rate of 77.81 ± 9.76% in the PLCNP + resection + PDT group. The median survival of the animals was 92 days, compared with 52 days for the PLNP + resection + PDT group and 34 days in the group receiving saline solution. Furthermore, 42.86% of mice treated with PLCNP + resection + PDT survived for more than 100 days.

Jiang et al. also achieved marked inhibition of tumour growth. Ref. [20] for SOP MN. In the subcutaneous U87-MG model, the use of PDT alone led to a reduction in tumour growth of approximately 16 per cent, whilst combining the photodynamic module with BSA–MnO_2_ increased this effect to approximately 49 per cent. The inclusion of the PROTAC component resulted in tumour growth inhibition of approximately 60 per cent, whilst the complete SOP MN nanoplatform achieved approximately 86 per cent tumour growth inhibition following irradiation. This latter group was also characterised by the longest median survival. In the orthotopic GBM model, a significant reduction in bioluminescent signal and tumour volume was observed, and complete tumour elimination was noted in two animals. The therapy also significantly prolonged survival compared with the control group.

The efficacy of rGOQD/IR820/MnO_2_/Q/CPP in the U87 orthotopic model was demonstrated by Dash et al. [21]. The most pronounced inhibition of tumour progression was observed following the use of the complete nanoplatform and radiotherapy, as evidenced by a reduction in the bioluminescent signal compared with the control groups. Median survival increased from 16 days in the PBS group to 21 days following treatment with rGOQD/IR820/CPP combined with irradiation, and to 26 days in the rGOQD/IR820/MnO_2_/Q/CPP group combined with irradiation. The corresponding mean survival times were 16.0 ± 0.9, 20.6 ± 1.1 and 26.0 ± 1.6 days, respectively.

In the study by Zhao et al. [25] The FBFO@HM@aOPN nanoplatform demonstrated potent anti-tumour activity in the CT2A orthotopic model. Bioluminescent imaging confirmed significant inhibition of tumour growth, whilst histological analysis revealed a reduction in Ki-67 expression in the tumour tissue. Particularly favourable results were obtained when FBFO@HM@aOPN was combined with an anti-PD-1 antibody. The combination therapy led to a greater reduction in the bioluminescent signal than either method used alone, and all mice in this group survived until day 100 of observation. The authors also demonstrated increased resistance to re-implantation of tumour cells following combination therapy.

Hu et al. [19] demonstrated that Pt@PCN-222(Mn)-PEG inhibited GBM growth in the orthotopic U87-Luc model. The most pronounced reduction in the bioluminescent signal was observed in the Pt@PCN-222(Mn)-PEG + Laser group, which also exhibited the lowest tumour mass and the most favourable survival curve among the groups compared.

Zhang et al. [27] confirmed the anti-tumour activity of TTTMN NPs-Ang2 in both the subcutaneous and orthotopic GBM models. In the subcutaneous model, the most effective inhibition of tumour growth was achieved following the use of the nanoplatform and light-guided radiation delivery, with a significant advantage over the control groups (*p* < 0.001). In the intracranial model, the same strategy resulted in a marked reduction in relative bioluminescence intensity compared with external irradiation and the control groups. Furthermore, an increase in the number of TUNEL-positive cells and a reduction in Ki-67 expression were observed in tumour tissues.

In the case of LTPA, Zhang et al. [28] demonstrated significant inhibition of orthotopic GBM growth following the application of the nanoplatform and irradiation. Bioluminescent imaging confirmed a significant reduction in tumour progression compared with the control group and the LTP nanoplatform, as reflected in the quantitative signal analysis. Histopathological results indicated severe damage to tumour cells following treatment.

Jiang et al. [24] They also achieved significant tumour regression following the administration of Bi_2_S_3_/Ti_3_C_2_-TPP in the subcutaneous U251 model. In contrast to the control groups, in which further tumour growth was observed, the complete nanoplatform, following irradiation, led to a complete halt in tumour growth. No recurrence was observed during the two-week follow-up period. H&E and TUNEL histopathological examinations confirmed extensive damage to the tumour tissue, the greatest among the treatment options compared.

Zou et al. [29] demonstrated the efficacy of JSK@PEG-IR820 in a subcutaneous U87-MG model. Following irradiation, the complete nanoplatform led to a complete inhibition of tumour growth, whilst further progression was observed in the control groups. Histopathological examinations confirmed extensive damage to the tumour tissue, and TUNEL and Ki-67 assays demonstrated, respectively, increased cell death and reduced proliferation.

Li et al. [23] They assessed the efficacy of Fe_3_O_4_@PDA@Ru in a subcutaneous U87-MG model. The combined use of the nanoplatform and light irradiation led to a significant inhibition of tumour growth compared with the control group and the nanoplatform without light activation (*p* < 0.05). After 14 days of observation, the group receiving combination therapy had the smallest tumour volume and mass. H&E staining revealed reduced tumour cell density and extensive areas of necrosis.

In the case of RhB-MNPs [30] and H-CeO_2_@Ce6@PDA@GOx [22]. The available findings on therapeutic efficacy relate to the cellular studies described in the previous section of this chapter.

Taken together, the studies presented demonstrate that the analysed nanoplatforms enable a significant reduction in the viability and proliferation of GBM cells, an increase in cell death, and the inhibition of tumour growth. In some studies, complete tumour regression, a reduction in recurrence and a significant prolongation of animal survival were also achieved. Particularly favourable results were obtained following the use of comprehensive multimodal regimens combining PDT with additional therapeutic components (Table 2).

### 5.3. Mechanistic Interactions and Potential Trade-Offs in Combination Therapies

The therapeutic effects of multifunctional nanoplatforms depend not only on the individual activity of their components but also on the interactions between photodynamic reactions, oxygen metabolism, redox regulation and additional mechanisms of cell death. These interactions may be mutually reinforcing, but they may also involve competition for reaction substrates or activation of opposing biological responses. Consequently, improved antitumour efficacy following combined treatment should not automatically be interpreted as evidence of mechanistic synergy.

A particularly important interaction concerns PDT and ferroptosis. Ferroptosis is driven by the accumulation of oxidised membrane phospholipids, whereas glutathione peroxidase 4 (GPX4), using reduced glutathione (GSH), limits this process by reducing phospholipid hydroperoxides. PDT-derived ROS may initiate lipid peroxidation, while GSH depletion or impaired GPX4 activity may decrease the capacity of cells to eliminate the resulting oxidation products. Experimental evidence supporting such interactions was provided by Shui et al. [121], who demonstrated PDT-induced lipid peroxidation and a ferroptosis-like cell death pathway, and by Kojima et al. [122], who observed enhanced antitumour effects when PDT was combined with ferroptosis-inducing agents. In the context of GBM, Li et al. [23] reported decreased intracellular GSH, reduced GPX4 activity and increased lipid peroxidation following treatment with Fe_3_O_4_@PDA@Ru under irradiation. These findings are consistent with the involvement of ferroptosis-associated mechanisms; however, the concurrent photodynamic, photothermal and endoplasmic reticulum stress responses complicate attribution of the therapeutic outcome to a single pathway.

The relationship between oxygen generation and ferroptosis requires particular consideration. Increasing O_2_ availability may support both oxygen-dependent photodynamic reactions and oxidative lipid damage. Therefore, alleviation of hypoxia does not inherently oppose ferroptosis induction. Nevertheless, the final response depends on the balance between lipid oxidation and antioxidant protection, as well as the intracellular distribution of oxygen, iron and susceptible phospholipids. In addition, oxygen-generating and radical-producing catalytic pathways may compete for common substrates. For example, catalase-like H_2_O_2_ disproportionation produces O_2_, whereas Fe^2+^-mediated Fenton chemistry consumes H_2_O_2_ to generate hydroxyl radicals. Preferential utilisation of H_2_O_2_ in one pathway could therefore restrict its availability for the other, depending on catalytic kinetics, local pH and substrate concentrations. A related limitation concerns glucose oxidase-containing systems, such as H-CeO_2_@Ce6@PDA@GOx, in which glucose oxidation consumes O_2_ while producing H_2_O_2_ that may subsequently undergo catalase-like decomposition. The coexistence of these reactions does not establish a positive net oxygen balance, and their combined effect on PDT must be determined experimentally.

Other therapeutic combinations introduce additional interactions. Photothermal heating may enhance cellular damage and, under some conditions, influence perfusion and oxygen delivery, but these effects cannot be assumed without direct measurements. In rGOQD/IR820/MnO_2_/Q/CPP, quercetin was incorporated to interfere with heat-shock responses and enhance photothermal susceptibility, whereas MnO_2_ was intended to increase local oxygen availability for PDT [21]. In the SOP MN system, the oxygen-generating module supports a photodynamic process that also mediates cleavage of the photosensitive linker in the OT7 prodrug, coupling light-dependent ROS formation to the release of a complementary therapeutic agent [20]. These examples illustrate how different components may act through interconnected mechanisms, although the individual contributions of these interactions have not always been independently quantified.

Establishing genuine synergy or antagonism requires experimental designs that distinguish the effects of individual therapeutic modalities from those of their combination. Future studies should therefore compare treatment components under defined oxygen concentrations and include measurements of local oxygen availability, H_2_O_2_ consumption, GSH/GSSG balance, GPX4 activity and lipid peroxidation. Where ferroptosis is proposed, functional inhibition or rescue experiments using ferroptosis inhibitors and iron chelators would strengthen mechanistic attribution. Appropriate factorial treatment designs and quantitative interaction analyses would further help determine whether combined effects exceed those expected from independent mechanisms. Such approaches are essential for distinguishing complementary therapeutic activity from genuine mechanistic synergy and for determining whether oxygen-modulating strategies enhance or constrain additional oxidative pathways in GBM.

## 6. Biosafety and Translational Safety Considerations

### 6.1. Intracranial Light Delivery and Photodynamic Dosimetry

An additional major limitation of nanoparticle-mediated PDT in glioblastoma is the delivery of an adequate light dose to intracranial tumour tissue. Although nanoplatforms can improve photosensitiser delivery, modulate oxygen availability and promote alternative photochemical pathways, these properties do not overcome the fundamental requirement for effective photoactivation within the target region. Light propagation in brain tissue is affected by absorption and scattering, producing spatially heterogeneous irradiation and limiting the depth at which therapeutically relevant photon densities can be achieved. These limitations are particularly important in GBM because of its infiltrative growth, irregular tumour boundaries and proximity to functionally critical brain structures. Although longer excitation wavelengths may offer improved tissue penetration compared with shorter visible wavelengths, penetration depth remains dependent on tissue optical properties and irradiation geometry. Consequently, the use of near-infrared excitation does not, by itself, establish adequate illumination of a deeply located intracranial tumour.

One approach to overcoming these limitations involves direct light delivery through optical fibres. Among the nanoplatforms analysed in this review, Zhang et al. [27] investigated TTTMN NPs-Ang2 in combination with a modified optical fibre introduced into the tumour. The authors demonstrated enhanced phototherapeutic effects in experimental GBM models, including orthotopic tumours, illustrating the feasibility of combining nanoparticle-mediated Type I PDT with local intracranial illumination. Importantly, this approach addresses two distinct limitations: the photosensitiser is designed to maintain radical-associated photochemical activity under reduced oxygen availability, whereas the optical fibre improves access of the activating light to the tumour. Nevertheless, these findings were obtained in preclinical models and do not establish the safety, reproducibility or effectiveness of the same procedure in human GBM.

The clinical feasibility of intracranial light delivery has also been investigated using conventional photosensitising approaches. Two principal strategies are stereotactically guided interstitial PDT, in which cylindrical optical diffusers are positioned within the tumour, and intraoperative PDT, in which the walls of a resection cavity are illuminated after surgical removal of the tumour mass. Quach et al. [123] investigated interstitial PDT using 5-aminolevulinic acid-induced protoporphyrin IX and stereotactically implanted cylindrical diffusers delivering light at 635 nm in patients with newly diagnosed GBM. A different approach was evaluated in the INDYGO clinical study, in which a balloon-based diffuser enabled illumination of the surgical cavity following tumour resection. In the long-term follow-up reported by Peciu-Florianu et al. [124], the irradiation protocol delivered a total fluence of 200 J/cm^2^ at the balloon surface, divided into five fractions separated by 2 min intervals without irradiation. These studies demonstrate the technical feasibility of delivering therapeutic light within the human cranial cavity. However, their findings concern conventional 5-ALA-mediated PDT and cannot be directly extrapolated to nanoparticle-based systems with different photosensitisers, excitation wavelengths, tissue distributions and photochemical properties.

Importantly, the amount of light emitted by a source is not equivalent to the dose absorbed by the photosensitiser within the tumour. Effective photodynamic exposure depends on wavelength-dependent light attenuation, spatial fluence distribution, photosensitiser concentration and localisation, and local oxygen availability during irradiation. Finlayson et al. [125] used Monte Carlo radiative transport modelling to evaluate intracavitary PDT for GBM and demonstrated that modifying light dose and irradiation parameters can influence predicted tumour cell damage while simultaneously increasing the risk of excessive tissue heating. These findings emphasise the need for treatment-specific optical dosimetry rather than reliance solely on nominal laser power, exposure duration or fluence measured at the irradiation source. Fractionated illumination may allow partial oxygen replenishment between irradiation periods, but its effects depend on tissue perfusion, oxygen consumption and the characteristics of the photosensitiser, and therefore require experimental validation.

For nanoparticle-mediated PDT, future translational studies should integrate nanoplatform biodistribution with patient-specific planning of light delivery. Particular attention should be paid to selecting appropriate optical access routes, determining the spatial distribution of fluence within heterogeneous tumour tissue, evaluating the coverage of infiltrative tumour margins and limiting phototoxic or thermal damage to surrounding healthy brain structures. Experimental studies should also distinguish between light delivery to the tumour surface and effective illumination of deeper tumour regions. Ultimately, successful clinical translation will require coordinated optimisation of nanoplatform localisation, photochemical activity, oxygen availability and intracranial light dosimetry rather than independent improvement of any single component.

### 6.2. Biosafety and Long-Term Safety Considerations

The safety assessment of the analysed nanoplatforms primarily covered cytotoxicity towards non-cancerous cells, haemocompatibility, haematological and biochemical parameters, and histopathological examinations of major organs. Most publications indicated that the materials used were well tolerated within the dose ranges and observation periods studied. However, the scope of the analyses carried out varied, and the results obtained did not always allow for a comprehensive assessment of the safety of the entire therapeutic procedure.

Particularly significant data were provided by Chen et al. [15], who assessed the effect of PLCNP on primary neurons, including after irradiation, and carried out a haemolysis test, showing a haemolysis rate of less than 1 per cent. Biocompatibility studies on non-cancerous cells were also carried out by Dash et al. [21], using 3T3 fibroblasts, Li et al. [23], using HEK293T cells, and Süngü Akdogan et al. [22], using the L929 cell line. In addition, haemocompatibility was analysed for, amongst others, Bi_2_S_3_/Ti_3_C_2_-TPP and TTTMN NPs-Ang2. Although these results provide information on the tolerability of nanomaterials, studies conducted on fibroblast and renal cell lines do not replace an assessment of their safety with regard to normal cells of the central nervous system, including neurons, astrocytes and microglia, which may exhibit different responses to exposure to nanoparticles [126,127]. Apart from the study by Chen et al., direct assessment of the safety of phototherapy on normal neurons was limited, whilst the effects of the analysed nanoplatforms on astrocytes, oligodendrocytes and microglia were not systematically characterised in most studies.

In in vivo studies, the most commonly monitored parameters were the animals’ body weight, blood parameters and histopathological changes in the heart, liver, spleen, lungs and kidneys. Such analyses were carried out, amongst others, for PLCNP, Pt@PCN-222(Mn)-PEG, TTTMN NPs-Ang2, Bi_2_S_3_/Ti_3_C_2_-TPP and FBFO@HM@aOPN [15,19,27]. No clear signs of systemic toxicity were observed during the periods under review. Some of the studies also included biodistribution analyses. For example, Jiang et al. determined the levels of Bi and Ti in organs using the ICP-MS method, whilst Zou et al. [29] demonstrated the accumulation of JSK@PEG-IR820 in the liver, supplementing the safety assessment with tests of liver and kidney function parameters. However, these measurements do not provide a complete picture of the fate of nanomaterials in the body, for which long-term tissue retention, the degradation kinetics of individual components, the identification of metabolites, and the determination of the pathways and extent of elimination are also important [128,129]. These parameters were not comprehensively characterised in the studies analysed, which is of particular significance in the case of multi-component nanoplatforms containing metals and inorganic materials that are difficult to biodegrade.

Neurological safety remains a significant gap, as GBM therapy must take into account the risk of damage to healthy brain tissue resulting from the non-selective action of ROS, local overheating and the potential neurotoxicity of nanomaterials and their degradation products [126,130,131]. The studies analysed did not present a comprehensive assessment of long-term neurological consequences, covering cognitive functions, motor skills, the integrity of neurons and synapses, and chronic neuroinflammation. Strategies involving the interstitial delivery of radiation via optical fibres, the local implantation of microneedles and intense photothermal effects also require further safety assessment. In the case of these methods, consideration must be given not only to the toxicity of the nanomaterials themselves, but also to the potential consequences of the mechanical impact of the implanted components on brain tissue and the possibility of thermal damage to neighbouring neural structures [131,132,133]. Furthermore, immunomodulatory nanoplatforms such as FBFO@HM@aOPN warrant particular attention, as the induction of an anti-tumour immune response does not rule out the occurrence of adverse inflammatory reactions in the central nervous system. The significance of this potential risk is supported by reports of neurological complications associated with immunotherapy and studies indicating the involvement of glial cell activation in the neurotoxicity of nanomaterials [126,134]. This does not, however, mean that similar adverse effects have not been demonstrated for FBFO@HM@aOPN, but it does highlight the need for a separate assessment of these.

Ultimately, the results to date indicate favourable short-term tolerability for many of the nanoplatforms analysed; however, the scope of the available safety studies remains insufficient for a full assessment of their translational potential. The priority for further work should be long-term studies of neurotoxicity and systemic toxicity, quantitative characterisation of the degradation and elimination of nanomaterials, assessment of the safety of repeated administration, and determination of the impact of the complete phototherapeutic procedure on healthy brain tissue.

## 7. Conclusions

The analysis of 12 original experimental studies highlights the potential of nanoparticle-based strategies to address hypoxia-associated limitations of photodynamic therapy in glioblastoma. The investigated nanoplatforms employ two complementary approaches: increasing local oxygen availability through catalytic, chemical or photocatalytic processes, and promoting photochemical pathways with reduced dependence on molecular oxygen. However, the critical comparison of available evidence demonstrates that oxygen-generating capacity, improved oxygenation of the biological environment and preservation of photodynamic activity under hypoxic conditions represent distinct outcomes that cannot be considered interchangeable. In particular, oxygen production demonstrated in simplified experimental systems does not necessarily translate into increased oxygen availability in tumour tissue, where substrate limitations and competing oxygen-consuming processes may substantially influence the overall oxygen balance.

The evidence supporting sustained photodynamic activity under hypoxia remains heterogeneous. Although several nanoplatforms demonstrated ROS-associated responses and photocytotoxicity under experimentally induced oxygen deprivation, the interpretation of these findings is frequently limited by incomplete control of oxygen concentrations, the nonspecificity of ROS probes and the contribution of additional therapeutic mechanisms. Importantly, type I photochemical pathways do not necessarily eliminate oxygen dependence, particularly when superoxide generation is involved. Consequently, while the preservation of PDT-associated activity has been demonstrated for selected systems at the cellular level, direct quantitative evidence of maintained photochemical efficiency within hypoxic regions of intracranial GBM remains insufficient.

The therapeutic studies further demonstrate that multifunctional nanoplatforms can substantially inhibit tumour growth and, in several orthotopic models, prolong animal survival. Nevertheless, these outcomes frequently reflect the combined effects of PDT, photothermal therapy, redox modulation, targeted protein degradation or immunomodulation, rather than the isolated contribution of photodynamic reactions. The available preclinical results are therefore encouraging but do not establish the extent to which overcoming oxygen limitations independently determines therapeutic efficacy.

Future investigations should prioritise controlled comparisons of photochemical activity and cytotoxicity at defined oxygen concentrations, direct and spatially resolved measurements of intratumoural oxygenation, and experimental separation of PDT-mediated effects from accompanying therapeutic mechanisms. These approaches should be complemented by studies addressing heterogeneous tumour microenvironments, reproducible light delivery, nanoplatform biodistribution and clearance, and long-term neurological and systemic safety. Ultimately, the translational value of increasingly complex nanoplatforms will depend not only on their ability to generate oxygen or reactive species, but also on demonstrating that these properties produce reproducible, selective and therapeutically meaningful photodynamic effects within the hypoxic microenvironment of glioblastoma.

## Figures and Tables

**Table 1:** Oxygen-related strategies, validation assay, and light exposure parameters.

Nanoplatform	Designed Strategy	Oxygen-Related Validation	PDT Under Low O_2_	Light Source and Exposure Conditions
Pt@PCN-222(Mn)-PEG	Pt catalase-like H_2_O_2_ decomposition supplies O_2_ to Mn-porphyrin PDT.	Dissolved-O_2_ increase in H_2_O_2_ solutions (1.25–10 µM); cellular [Ru(dpp)_3_]Cl_2_ signal in CoCl_2_-treated U87; orthotopic photoacoustic haemoglobin sO_2_.	No direct PDT ROS/photocytotoxicity comparison at defined physical low O_2_; CoCl_2_ is a hypoxia mimetic.	In vitro: Laser 660 nm; 2 W/cm^2^; 150 s; 300 J/cm^2^ (cellular PDT assays). In vivo: Laser 660 nm; 2 W/cm^2^; 150 s; 300 J/cm^2^ (reported regimen).
PLCNP	Acid-triggered CaO_2_ breakdown supplies H_2_O_2_/O_2_; porphyrin activation accompanies carrier disassembly.	pH-dependent oxygen-probe luminescence; microelectrode tissue pO_2_ (orthotopic GBM), with inconsistent group labels in Figure S14a.	GICs at 1% O_2_: CCK-8 after PDT; PLCNP vs. core-free PLNP difference in the specified SI comparison was not significant (*p* = 0.1714).	In vitro: Laser 660 nm; 0.1 W output over 0.32 cm^2^; 6 s; 1.875 J/cm^2^ (GIC PDT); 3–6 J/cm^2^ in a separate neural-cell safety assay. In vivo: Laser 660 nm; 0.1 W output over 0.32 cm^2^; 25 s; 7.81 J/cm^2^ (cranial window).
SOP MN	BSA–MnO_2_ converts H_2_O_2_ to O_2_; microneedles deliver the oxygen-generating and PPT7/PROTAC modules sequentially.	Dissolved-O_2_ measurement for BSA–MnO_2_ at 100 µM H_2_O_2_ (pH 6.5 vs. 7.4); reduced tumour HIF-associated staining.	U87-MG at 1% O_2_: ROS-associated probe signal, CCK-8 and cleaved caspase-3; probe identity inconsistent between main text and SI.	In vitro: Laser 671 nm; 100 mW/cm^2^; 1 min; 6 J/cm^2^ (U87-MG phototoxicity). In vivo: Laser 671 nm; 100 mW/cm^2^; 10 min; 60 J/cm^2^ (subcutaneous and orthotopic models).
rGOQD/IR820/MnO_2_/Q/CPP	MnO_2_/H_2_O_2_ reaction intended to supply O_2_ near IR820; quercetin supports PTT by modulating thermotolerance.	Gas bubble formation at 40 mM H_2_O_2_; cellular hypoxia-indicator signal and HIF-1α immunoassays.	No defined-O_2_ normoxia–hypoxia comparison of photochemical ROS or PDT-specific cytotoxicity.	In vitro: NIR laser 808 nm; 1 W/cm^2^; 3 min; 180 J/cm^2^ (U87 cell assays). In vivo: NIR laser 808 nm; 1 W/cm^2^; 5 min; 300 J/cm^2^ (orthotopic U87).
H-CeO_2_@Ce6@PDA@Gox	GOx consumes glucose/O_2_ and forms H_2_O_2_; CeO_2_ catalase-like activity recycles some O_2_ for Ce6-PDT.	H_2_O_2_ depletion kinetics (K_m_ ≈ 0.31 mM for H-CeO_2_@PDA); oxygen production of the complete GOx–CeO_2_ cascade not quantified.	No controlled low-O_2_ PDT comparison; the GOx–CeO_2_ reaction sequence does not imply net O_2_ generation.	In vitro: Red LED 650 nm; 0.8 W total output; 7 min (PDT); NIR laser 808 nm; 3.2 W/cm^2^; 5 min (PTT; 960 J/cm^2^). LED fluence not calculable from reported power. In vivo: Not investigated in vivo. Note: an inconsistent “3.2 mW/cm^2^” label appears in a photothermal figure, vs. 3.2 W/cm^2^ in methods.
Fe_3_O_4_@PDA@Ru	Ru(II) complex mediates oxygen-dependent photosensitization; polydopamine adds PTT.	^1^O_2_ generation assessed by TEMP-EPR/SOSG; intracellular oxidation and cytotoxicity after light exposure.	No defined-O_2_ normoxia–hypoxia comparison of ROS or photocytotoxicity.	In vitro: Laser 450 nm; 62.7 mW/cm^2^; 5 min; 18.81 J/cm^2^ (PDT); laser 808 nm; 7.8 W/cm^2^; 5 min; 2340 J/cm^2^ (PTT). In vivo: Laser 450 nm; 62.7 mW/cm^2^; 10 min; 37.62 J/cm^2^; plus laser 808 nm; 3.16 W/cm^2^; 10 min; 1896 J/cm^2^.
Bi_2_S_3_/Ti_3_C_2_-TPP	Photogenerated charge carriers enable proposed water oxidation/O_2_ production and radical-forming type-I routes; TPP targets mitochondria.	Oxygen-related photocatalytic studies; Image-iT hypoxia indicator; ROS assays in separate experiments.	Air vs. N_2_ comparison after irradiation (MTT); O_2_ concentration during this comparison not measured.	In vitro: NIR laser 808 nm; 1 W/cm^2^; 10 min; 600 J/cm^2^ (U251). In vivo: NIR laser 808 nm; 1 W/cm^2^; 10 min; 600 J/cm^2^ (subcutaneous model).
FBFO@HM@aOPN	Fe_3_O_4_@BiFeO_3_ photocatalytic O_2_ release from H_2_O_2_, type-I ROS and Fe-dependent Fenton-like oxidation.	Dissolved-O_2_ measurement with 100 µM H_2_O_2_; hypoxia-associated signal in CT2A 3D cultures.	Normoxia vs. nominal hypoxia: CCK-8 phototherapy comparison; hypoxic O_2_ level not defined and PDT cannot be isolated from other reactions.	In vitro: Laser 660 nm; 0.5 W/cm^2^; 10 min; 300 J/cm^2^ (CT2A assays). In vivo: Laser 660 nm; 10 min; irradiance / fluence not clearly specified for the in vivo treatment in the main text.
TTTMN NPs-Ang2	Donor–acceptor AIE photosensitizer promotes electron transfer and type-I radical pathways; Angiopep-2 supports delivery.	Relative DCFH-DA/DHR123 signals under normal and hypoxia-mimicking atmospheres; separate ESR evidence for radicals.	GL261: comparative ROS-associated fluorescence and CCK-8; AnaeroGen chamber with no measured medium O_2_.	In vitro: Laser 520 nm; 100 mW total output for cellular ROS assays (2 min) and through-fibre/collimator model assays; beam area not specified consistently; incident fluence not derived. In vivo: Laser 520 nm; nominal 100 mW/cm^2^ for 20 min via collimator or optical fibre; nominal 120 J/cm^2^ at output, not a measured intratumoural dose.
LTPA	Donor–acceptor LT photosensitizer promotes type-I electron transfer; Angiopep-2 delivery; parallel photothermal action.	Relative fluorescence signals for ROS under 21% and 2% O_2_ (U87).	U87: ROS-probe and CCK-8 comparison at 2% O_2_; additional PDT/PTT separation used cooling, with no verified O_2_ level.	In vitro: NIR laser 808 nm; 1 W/cm^2^; 10 min; 600 J/cm^2^ (U87). In vivo: NIR laser 808 nm; 1 W/cm^2^; 10 min; 600 J/cm^2^ (orthotopic U87).
JSK@PEG-IR820	Polymer-assisted type-I photochemistry plus GSH-triggered NO delivery, permitting ONOO^−^ formation.	ROS/NO/RNS indicators and cytotoxicity; related assays were not documented as matched O_2_-controlled comparisons.	U87-MG at 2% O_2_: MTT phototoxicity; no matched normoxia comparison or direct PDT-specific radical measurement.	In vitro: NIR laser 808 nm; 0.5 W/cm^2^; 8 min; 240 J/cm^2^ (U87-MG viability). In vivo: NIR laser 808 nm; 0.5 W/cm^2^; 8 min; 240 J/cm^2^ (subcutaneous U87-MG).
RhB-MNPs	Rhodamine B on natural melanin; type-I and type-II routes proposed without an O_2_-generating module.	Fluorescent oxidant detection and inhibition studies; no dedicated oxygen-generating component.	U87-MG: PDT response tested at 1% O_2_ with MTT; similar metabolic-viability response in normoxia.	In vitro: CoolLED pE-300 green LED, 550 nm; 97 mW/cm^2^; 5 min; 29.1 J/cm^2^ (U87-MG). In vivo: Not investigated in vivo.

Notes. Incident fluences (J/cm^2^) calculated as irradiance (W/cm^2^) × exposure time (s) where numerical irradiance and time were reported. Calculated fluence is not necessarily a measured intratumoural dose; “NR” indicates not reported or not directly calculable. Protocols shown are representative study-specific regimens, not universal parameters. For multiphase PDT/PTT, both irradiations are presented separately. Values and nomenclature should be checked again against primary papers before final submission.

**Table 2:** In vitro and in vivo antitumour outcomes, including reported IC_50_.

Nanoplatform	Reported IC_50_ (In Vitro)	Other In Vitro Outcomes	In Vivo Efficacy/Survival	Therapeutic Context
Pt@PCN-222(Mn)-PEG	NR	U87: concentration-dependent CCK-8 response after irradiation; Calcein-AM/PI and Annexin V/PI analyses.	Orthotopic U87-Luc: strongest bioluminescence reduction and most favourable survival curve for nanoparticle + laser.	PDT + Pt-mediated oxygenation/redox effects; no discrete PDT contribution quantified.
PLCNP	NR	GICs: post-PDT CCK-8 response; cell death-related readouts in separate assays.	Orthotopic GIC-derived GBM + resection/PDT: tumour elimination 77.81 ± 9.76%; median survival 92 d vs. 52 d (PLNP + resection/PDT) and 34 d (saline); 42.86% survived >100 d.	Resection plus PDT; survival results refer to the complete treatment regimen.
SOP MN	0.18 µg/mL (PPT7 + light); 0.09 µg/mL (PPT7 + BSA–MnO_2_ + light); CCK-8, hypoxic U87-MG.	U87-MG, 1% O_2_: CCK-8 IC_50_ 0.18 → 0.09 µg/mL (PPT7 + PDT vs. PPT7/BSA–MnO_2_ + PDT); cleaved caspase-3 increased.	Subcutaneous U87-MG: ~86% tumour growth inhibition for complete illuminated system; orthotopic GBM: marked tumour reduction, two tumour-free animals reported.	PDT + oxygen-supply module + light-triggered BRD4-degrading PROTAC.
rGOQD/IR820/MnO_2_/Q/CPP	NR	U87: lower MTT metabolic viability and higher apoptosis/necrosis indicators after combined light treatment.	Orthotopic U87: median survival 16 d (PBS), 21 d (rGOQD/IR820/CPP + light), 26 d (complete platform + light).	PDT + PTT + quercetin; combined effect cannot be assigned to PDT alone.
H-CeO_2_@Ce6@PDA@GOx	NR	T98G: 10.6–13.1% relative MTT viability at 1–2 mg/mL with combined ST/CDT/PDT/PTT; AO/PI staining.	Not investigated in an animal GBM model.	Enzyme-mediated starvation + CDT + PDT + PTT.
Fe_3_O_4_@PDA@Ru	15.3 µg/mL (0.0153 mg/mL); MTT, combined 450 + 808 nm exposure.	U87-MG: MTT IC_50_ ≈ 15.3 µg/mL following combined photoactivation; Calcein-AM/PI and flow-cytometry analyses.	Subcutaneous U87-MG: lower tumour volume and mass after 14 d; histological damage reported.	Ru-mediated PDT + polydopamine-mediated PTT.
Bi_2_S_3_/Ti_3_C_2_-TPP	NR	U251: ~8.9% relative MTT viability following irradiation; 33.73% vs. 23.43% apoptosis/necrosis measures (TPP-targeted vs. untargeted).	Subcutaneous U251: tumour growth strongly suppressed; no regrowth reported during the 14-day observation.	Photocatalytic PDT-like chemistry + PTT + mitochondrial targeting.
FBFO@HM@aOPN	NR	CT2A: ~17% vs. ~40% CCK-8 relative viability in normoxia vs. nominal hypoxia; immunogenic-cell-death markers.	Orthotopic CT2A: all mice in complete-platform + anti-PD-1 group survived to day 100; enhanced response to rechallenge.	Photocatalysis/Fenton-like chemistry + anti-OPN immunomodulation + separate anti-PD-1 treatment.
TTTMN NPs-Ang2	NR	GL261: reduced CCK-8 viability following irradiation; Calcein-AM/PI and Annexin V/PI support cytotoxicity in other settings.	Subcutaneous and orthotopic GBM: improved tumour suppression with fibre-optic irradiation; decreased Ki-67 and increased TUNEL in tumours.	Type-I PDT with local fibre-optic illumination.
LTPA	NR	U87: CCK-8 response to light-mediated PDT/PTT; Calcein-AM/PI and Annexin V/PI analyses.	Orthotopic U87: attenuated bioluminescent tumour progression after LTPA + irradiation.	Combined PDT/PTT; cooling-based PDT isolation is not a direct in vivo estimate.
JSK@PEG-IR820	1.4 µg/mL (JSK@PEG-IR820 + light); MTT, U87-MG.	U87-MG: IC_50_ ≈ 1.4 µg/mL; Annexin V/PI apoptosis 43.3% vs. 31.1% (JSK) and 20.9% (PEG-IR820 + light).	Subcutaneous U87-MG: tumour growth arrested in the observation window; histological tumour injury.	PDT/PTT-associated oxidative effects plus NO/ONOO^−^ chemistry.
RhB-MNPs	NR	U87-MG: MTT relative viability 49 ± 8% after irradiation at 1% O_2_.	Not investigated in an animal GBM model.	Rhodamine-based PDT without an additional therapeutic modality.

Notes. NR: no numerical IC_50_ reported in the original experiment examined; it does not imply the nanoplatform lacked cytotoxicity. IC_50_ is only listed when explicitly stated or calculated by authors; it must not be inferred from a single viability data point. IC_50_ values obtained using different assays, photosensitizer dose units, irradiation regimens and combination treatments are not directly comparable. Efficacy results are transcribed for the corresponding arms and should not be interpreted as the contribution of PDT alone.

## Data Availability

No new data were created or analyzed in this study.

## Abbreviations

Abbreviation	Definition
AIE	Aggregation-induced emission
ApoE3	Apolipoprotein E3
BBB	Blood–brain barrier
BBTB	Blood–brain tumour barrier
BET	Bromodomain and extra-terminal
BRD4	Bromodomain-containing protein 4
BSA	Bovine serum albumin
CCK-8	Cell Counting Kit-8
CDT	Chemodynamic therapy
CPP	Cell-penetrating peptide
DCFH-DA	2′,7′-Dichlorodihydrofluorescein diacetate
DHE	Dihydroethidium
DHR123	Dihydrorhodamine 123
EPR	Electron paramagnetic resonance
ESR	Electron spin resonance
FITC	Fluorescein isothiocyanate
GBM	Glioblastoma
GICs	Glioma-initiating cells
GOx	Glucose oxidase
GPx1	Glutathione peroxidase 1
GSH	Glutathione
HIF-1α	Hypoxia-inducible factor 1 alpha
HSP70	Heat shock protein 70
HPF	Hydroxyphenyl fluorescein
ICP-MS	Inductively coupled plasma mass spectrometry
ISC	Intersystem crossing
LDLR	Low-density lipoprotein receptor
LRP1	Low-density lipoprotein receptor-related protein 1
MOF	Metal–organic framework
NO	Nitric oxide
OMVs	Outer membrane vesicles
OPN	Osteopontin
PBS	Phosphate-buffered saline
PDA	Polydopamine
PDT	Photodynamic therapy
PEG	Polyethylene glycol
PI	Propidium iodide
PROTAC	Proteolysis-targeting chimera
PTT	Photothermal therapy
ROS	Reactive oxygen species
SOSG	Singlet Oxygen Sensor Green
TPP	Triphenylphosphonium
TUNEL	Terminal deoxynucleotidyl transferase dUTP nick-end labeling
